# Targeting endoplasmic reticulum stress and nitroso-redox imbalance in neuroendocrine prostate cancer: the therapeutic role of nitric oxide

**DOI:** 10.1038/s41420-025-02774-5

**Published:** 2025-11-06

**Authors:** Fakiha Firdaus, Osmel Companioni Napoles, Raul Ariel Dulce, Anusha Edupuganti, Surinder Kumar, Khushi Shah, Salih Salihoglu, Derek J. Van Booven, Richard Gasca, David B. Lombard, Joshua M. Hare, Fangliang Zhang, Rehana Qureshi, Himanshu Arora

**Affiliations:** 1 Department of Urology, Miller School of Medicine, University of Miami, Miami, FL USA; 2 The Interdisciplinary Stem Cell Institute, Miller School of Medicine, University of Miami, Miami, FL USA; 3 Department of Pathology & Laboratory Medicine, Miller School of Medicine, and Sylvester Comprehensive Cancer Center, University of Miami, Miami, FL USA; 4 Miami VA Healthcare System, Miami, FL USA; 5 Department of Industrial and Systems Engineering, University of Miami, Coral Gables, FL USA; 6 John P Hussman Institute for Human Genomics, Miller School of Medicine, University of Miami, Miami, FL USA; 7 Rosalind Franklin University of Medical and Science, North Chicago, IL USA; 8 Sylvester Comprehensive Cancer Centre, Miami, FL USA; 9 Department of Medicine, Cardiology Division, Miller School of Medicine, University of Miami, Miami, FL USA; 10 Department of Molecular & Cellular Pharmacology, Miller School of Medicine, University of Miami, Miami, FL USA

**Keywords:** Prostate cancer, Nitrosylation

## Abstract

Neuroendocrine prostate cancer (NEPC) is an aggressive and therapy-resistant subtype of prostate cancer characterized by high levels of endoplasmic reticulum (ER) stress and metabolic dysregulation. The subsequential metabolic adaptations in the cancer cells reinforce survival mechanisms that contribute to therapy resistance and metastasis. The oncogenic driver neuroblastoma-derived MYC (MYCN) exacerbates ER stress by increasing calcium ion efflux from the ER into mitochondria, promoting glycolytic and oxidative stress. Here, we demonstrate that nitric oxide (NO) signaling is dysregulated in NEPC, thus allowing impaired S-nitrosylation of MYCN and uncontrolled ER stress. We show that exogenous NO supplementation restores MYCN S-nitrosylation at Cys4, Cys186, and Cys464. This re-establishment significantly reduces ER stress markers, inhibits the unfolded protein response (UPR), and suppresses NEPC cell proliferation and colony formation in vitro. In an orthotopic NEPC murine model, NO treatment led to a substantial reduction in tumor burden and metastasis to the liver and brain, with corresponding decreases in chromogranin and synaptophysin expression. Additionally, NO supplementation attenuated glycolytic stress by limiting calcium-mediated mitochondrial dysfunction and modulating metabolic pathways. Our findings uncover a direct mechanistic link between MYCN-driven ER stress and NEPC progression and highlight NO supplementation as a potential therapeutic strategy to counteract lineage plasticity and metabolic adaptations in NEPC. These results provide a compelling rationale for further investigation into NO-based therapies as a novel intervention for NEPC, a cancer subtype with limited treatment options and poor prognosis.

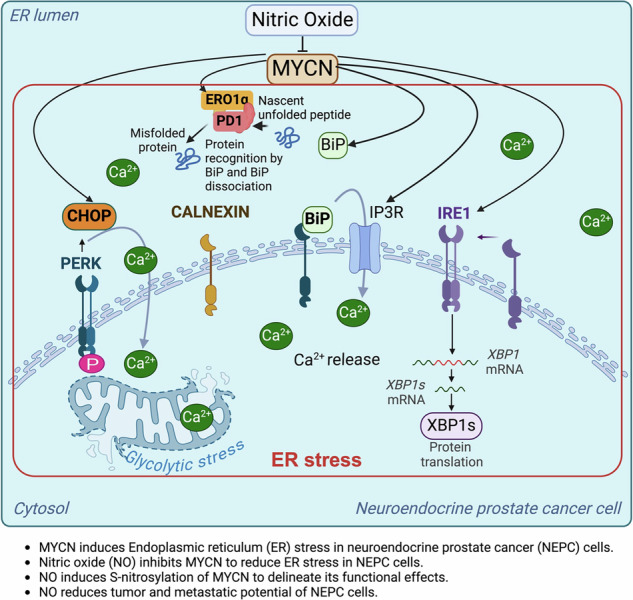

## Introduction

Neuroendocrine prostate cancer (NEPC) is an aggressive and highly therapy-resistant subtype of prostate cancer, contributing to up to 25% of prostate cancer-related deaths [1, 2]. NEPC typically arises as an adaptive response to androgen deprivation therapies (ADT) in late-stage, castration-resistant prostate cancer (CRPC), a process that is often marked by transdifferentiation from adenocarcinoma to neuroendocrine phenotype [3, 4]. This transformation, known as lineage plasticity, enables cancer cells to evade many conventional therapies, such as androgen receptor (AR)-targeted therapies and taxane-based chemotherapies, rendering them highly resistant and contributing to rapid disease progression [5, 6]. Current treatment strategies for NEPC, including chemotherapies in combination with taxanes or etoposide, have shown limited success in improving patient outcomes [5, 6]. Ongoing clinical trials exploring combinations of chemo-immunotherapies, such as nivolumab with cabazitaxel and carboplatin, have yet to demonstrate significantly enhanced efficacy [5, 6]. A key factor driving NEPC progression is the ability of cancer cells to tolerate severe metabolic and environmental stressors through adaptation to endoplasmic reticulum (ER) stress [7, 8]. The ER stress response, or the unfolded protein response (UPR), is activated by the accumulation of misfolded or unfolded proteins in the ER, triggering signaling pathways aimed at restoring cellular homeostasis [7, 8]. However, prolonged or unresolved ER stress can lead to mitochondrial dysfunction, oxidative stress, and activation of pro-survival pathways, all of which promote tumor growth, therapy resistance, and metastasis [9–12]. In NEPC, heightened ER stress not only supports cell survival under metabolic duress but also promotes the transition from an androgen-dependent adenocarcinoma to an androgen receptor (AR)-independent neuroendocrine phenotype [13–16]. This transition is tightly regulated by oncogenic drivers such as neuroblastoma-derived MYC (MYCN), which play a pivotal role in reprogramming gene expression and promoting metabolic reorganization to meet the increased biosynthetic demands of aggressive cancer cells [13, 17].

MYCN has a significant role in NEPC, where its overexpression drives epigenetic reprogramming, enhances UPR activation, and fosters lineage plasticity [13, 17]. Recent studies have demonstrated that MYCN promotes ER stress by increasing calcium ion efflux from the ER, which is then absorbed by mitochondria, further increasing glycolytic and oxidative stress [13, 17]. These stress adaptations create a feedback loop that supports the aggressive behavior of cancer cells, contributing to therapy resistance and metastatic potential. Notably, our previous studies have shown that S-nitrosylation, a post-translational modification that involves the covalent attachment of a nitric oxide (NO) group to the side chain of cysteine residues, plays a critical role in regulating protein function and modulating cancer cell signaling [18]. However, in high-grade PCa, the production of NO is significantly impaired due to dysregulation in endothelial nitric oxide synthase (eNOS) activity [18]. This may prevent the S-nitrosylation of many proteins including MYCN and contribute to the persistence of ER stress.

Given the central role of ER stress and MYCN in driving NEPC progression, we hypothesized that exogenous supplementation of NO could restore the S-nitrosylation of MYCN, thereby reducing ER stress and mitigating the aggressive behavior of NEPC cells. Nitric oxide has been shown to have a range of cytoprotective effects, including reducing oxidative stress and restoring normal cellular homeostasis. By reinstating the S-nitrosylation of MYCN, NO-based therapies could re-regulate key signaling pathways that promote ER stress, inhibit transdifferentiation, and reduce the overall tumor burden in NEPC.

In this study, we explored the molecular mechanisms by which ER stress and MYCN contribute to the pathogenesis of NEPC and investigated the therapeutic potential of NO supplementation in re-regulating these processes. Using a combination of in vitro cell models and an in-vivo orthotopic murine xenograft model, we demonstrated that NO treatment effectively restored MYCN S-nitrosylation, reduced ER stress markers, and inhibited the transdifferentiation of prostate adenocarcinoma cells to neuroendocrine phenotype. Moreover, we observed a significant reduction in tumor growth and metastasis following NO treatment, highlighting the potential of NO-based therapies as a novel strategy to combat therapy-resistant NEPC.

Together, our findings provide critical insights into the molecular mechanisms underlying NEPC progression and suggest that NO supplementation represents a promising therapeutic avenue for targeting ER stress and lineage plasticity in this aggressive cancer subtype.

## Results

### ER stress biomarkers correlate with prostate cancer progression and survival outcomes

**Fig. 1:**
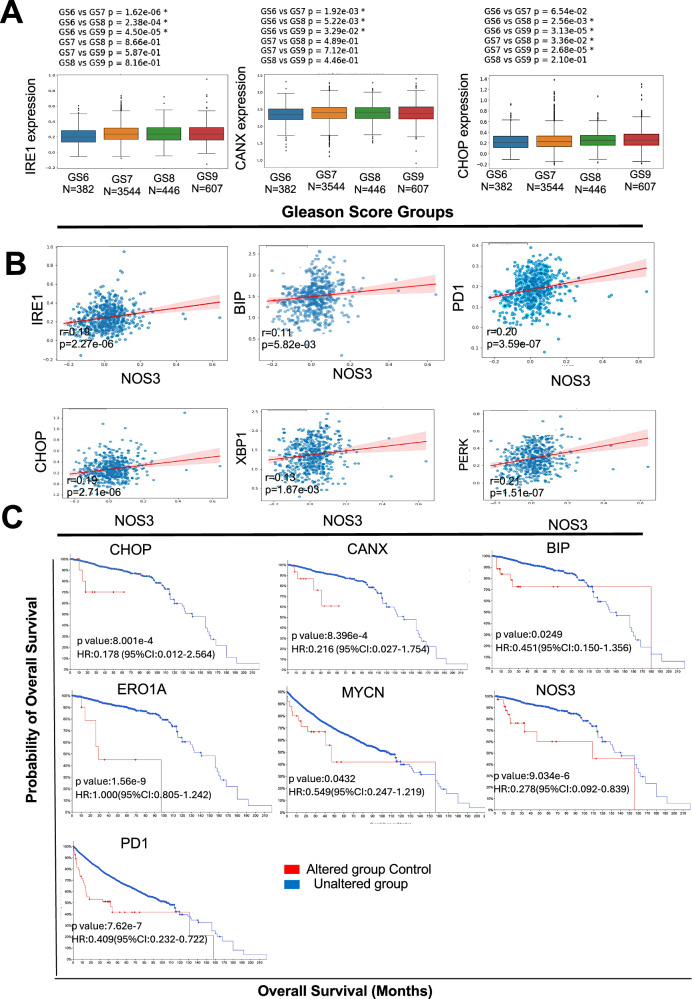
An enrichment analysis showing ER stress biomarkers correlate with prostate cancer (PCa) progression and survival outcomes. **A** The expression of ER stress markers in PCa patients was obtained from the Decipher Grid. The box plots span the 25th to 75th percentiles, and the expression is represented as log10log10 (reads per kilobase million, RPKM). The median (midline of box plots) and maximum and minimum (upper and lower bounds of whiskers) values are depicted. Significance was determined by a non-parametric Wilcoxon signed-rank test (two-sided), and *P* values correspond to the comparison between Gleason score groups with other subgroups of PCa patients. **B** The plots show a significant positive correlation between ER stress markers (IRE1, BiP, PDI, PERK, CHOP, and XBP1) with NOS3 in the Decipher Grid data. **C** Kaplan–Meier survival curves segregated by BiP, CHOP, CANX, ERO1A, MYCN, NOS3, and PDI expression. Overall survival is significantly diminished for individuals with high expression. *P* values were calculated using a log-rank (Mantel–Cox) test.

To investigate the relationship between ER stress biomarkers and PCa progression, RNA sequencing (RNAseq) data from 500 patients in the Cancer Genome Atlas (TCGA) prostate adenocarcinoma (PRAD) cohort and 4983 patients from the Decipher GRID database were analyzed. Although the Decipher GRID and TCGA databases primarily consist of PRAD samples, they remain valuable for studying the molecular landscape relevant to NEPC, considering the majority of the cases of NEPC evolve from adenocarcinoma, especially therapy-induced NEPC (t-NEPC), following androgen deprivation therapy (ADT) [19, 20]. Therefore, the patients were stratified based on their Gleason scores (GS) and clinical parameters such as age, PSA levels, and therapy history. In the TCGA cohort, the distribution included 52 noncancerous controls, 45 patients with GS6, 247 with GS7, 64 with GS8, 136 with GS9, and 4 with GS10. The Decipher GRID cohort comprised 382 patients with GS6, 3544 with GS7, 446 with GS8, and 607 with GS9. The differential expression analysis of these data revealed a significant upregulation of several key ER stress markers—IRE1 (ERN1), CANX (Calnexin), CHOP (DDIT3), XBP1, and PDI (protein disulfide isomerase-A1)(Fig. 1A, Supplementary Fig. 1) in high-grade tumors (GS ≥ 8), compared to lower-grade tumors and noncancerous controls (Fig. 1A, B). This upregulation was consistently observed across both the TCGA and Decipher GRID cohorts, suggesting that ER stress is a prominent feature of advanced PCa.

To explore the link between nitric oxide signaling and ER stress, we performed a correlation analysis between NOS3 (endothelial nitric oxide synthase) and ER stress markers. A significant positive correlation was observed between NOS3 and IRE1 (*r* = 0.19, *p* = 2.27e-06), BiP (*r* = 0.11, *p* = 5.82e-03), PDI (*r* = 0.20, *p* = 3.59e-07), XBP1 (*r* = 0.13, *p* = 1.67e-03), PERK (*r* = 0.21, *p* = 1.51e-07), and CHOP (*r* = 0.19, *p* = 2.71e-06) in the Decipher GRID dataset (Fig. 1B). These correlations suggest that NOS3 dysregulation is associated with increased ER stress in high-grade PCa, potentially contributing to tumor progression by amplifying the stress response.

To assess the clinical significance of ER stress markers in PCa, we conducted a survival analysis using the cBioPortal platform. Kaplan-Meier survival curves showed that high expression of BiP, CHOP, and IRE1 was associated with significantly reduced overall survival in PCa patients (Fig. 1C). For example, elevated CHOP expression was associated with a hazard ratio (HR) of 0.178 (*p* = 8.001e-4), while high CANX (Calnexin) expression yielded an HR of 0.216 (*p* = 8.396e-4). Additionally, increased PDI expression was correlated with poor survival outcomes (HR = 0.409, *p* = 7.62e-7). These findings suggest that elevated ER stress marker expression is linked to worse clinical outcomes, particularly in high-grade PCa patients.

### MYC increases the ER stress responses in PCa cells and these responses are correlated with increased nitroso-redox imbalance

**Fig. 2:**
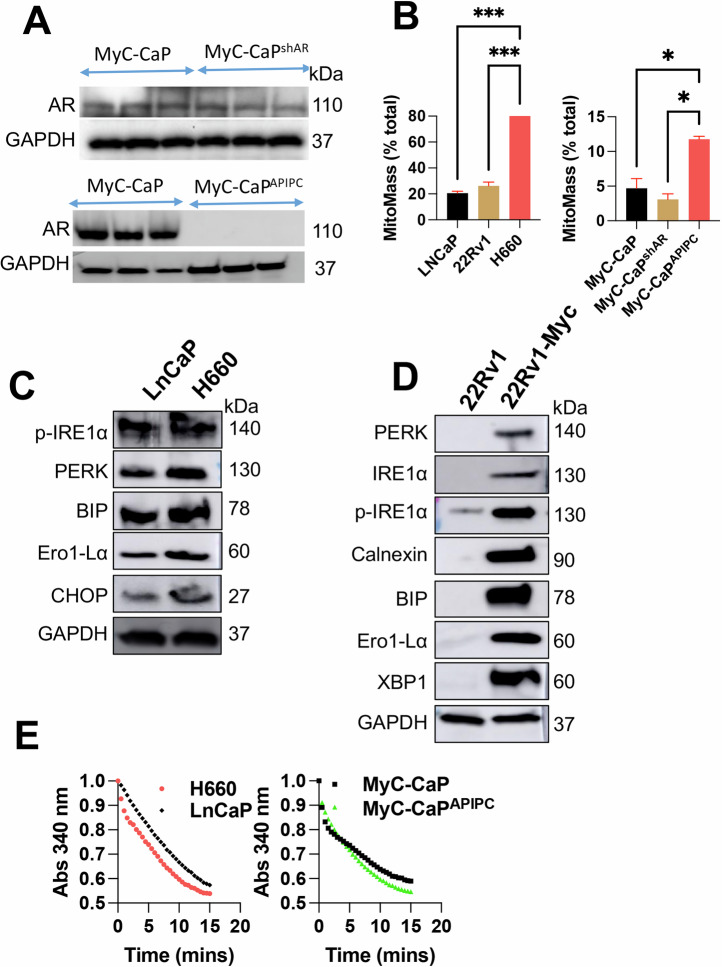
MYC enhances ER stress responses and nitroso-redox imbalance in PCa Cells. **A** Western blot analysis showing androgen receptor (AR) levels in MyCaP, MyCaPshAR, and MyCaPAPIPC cell lines. GAPDH serves as a loading control. **B** Bar graph depicting percent Mitomass in various PCa cell lines (LNCaP, 22Rv1, H660, MyCaP, MyCaPshAR, and MyCaPAPIPC). **C** Western blot comparing ER stress markers between LNCaP and H660 cell lines. GAPDH serves as a loading control. **D** Western blot showing ER stress marker expression in 22Rv1 cells with and without stable MYCC or MYCN overexpression. GAPDH serves as a loading control. **E** Bar graphs comparing GSNOR activity between LNCaP and H660 cells (left panel) and between MyCaP and MyCaPAPIPC cells (right panel). Data represent mean ± standard deviation from three independent biological replicates (*p* < 0.001, two-way ANOVA).

We developed test models of AR loss using MyC-CaP cells, an epithelial-like murine cell line that was originally developed from the prostate of a 16-month-old, male mouse with PCa. For this, MyC-CaP cells were subjected to the partial loss of AR (MyC-CaP^ShAR^) or the complete loss (MyC-CaP^APIPC^) using SMARTvector Inducible Lentiviral shRNA vector. Steps involved in the generation of MyC-CaP^APIPC^ from MyC-CaP^shAR^ are highlighted in the Supplementary Fig. 2A). MyC-CaP^APIPC^ cells showed complete AR absence (Fig. 2A) and increased MYCN expression, which suggests neuroendocrine phenotype formation (Supplementary Fig. 2B). To elucidate the relationship between nitroso redox imbalance and ER stress and to assess the involvement of mitochondrial dysfunction in this interplay, we used LNCaP, 22Rv1, H660, MyCAP, MyC-CaP^shAR^ and MyC-CaP^APIPC^ cells. The mitochondrial mass was quantified using fluorescent mitochondrial dyes, which provided insights into the bioenergetic health of the cells. We observed a significant increase in mitochondrial mass in cells representing high-grade PCa (Fig. 1B). The unfolded protein response (UPR) markers, such as IRE1 [21], BIP [22], Calnexin [23], CHOP [24], Ero1-La [25], and PERK [11] were examined to gauge ER stress levels using LNCaP and H660 cells. We observed a notable upregulation in these markers, particularly in H660 cells, indicating a heightened ER stress response in NEPC (Fig. 1C).

Further, we measured nitrate concentrations in these cell lines to estimate nitrosative stress (Supplementary Fig. 3). A negative correlation was established between nitrate concentrations (which were reduced) and the expression of Cysteine Sulfenic Acid (CSA) (which was increased) in our earlier study [18], underscoring the interconnection between nitrosative stress and hypoxic conditions in PCa. Next, to determine the impact of MYC on ER stress response elements, we generated 22Rv1 cells that stably overexpressed MYCC and MYCN. The rationale for over-expressing MYCC along with MYCN in 22Rv1 cells is the fact that CRPC cells do not biologically express MYCN, and doing so may not represent the typical stress responses seen in CRPC [26, 27]. Subsequent analyses revealed an exacerbation of ER stress markers, including BiP, Calnexin, Ero1-Lα, IRE1α, p-IRE1α, PERK, and XBP1, upon both MYCC and MYCN overexpression (Fig. 2D). These findings provide direct evidence of MYCN role in amplifying the malignant phenotype through the induction of ER stress.

Next, we confirmed that similar to CRPC [18], in NEPC cells (H660), diminished tetrahydrobiopterin to the 7,8-dihydrobiopterin ratio (BH4:BH2) ratio led to the destabilization of NOS subunits (uncoupling) and shift the enzymatic activity towards superoxide (O2-) generation [28–32]. This is reflected by a subsequent decrease in the activity of S-Nitrosoglutathione reductase (GSNOR) in proteins derived from H660 and MyC-CaP^APIPC^ cells compared to LNCAP and MyC-CaP cells (Fig. 2E). This decrease in GSNOR activity in high-grade PCa cells implies an accelerated depletion rate of S-Nitrosoglutathione (GSNO) in NEPC [18]. Together, these results conclusively demonstrate that MYC increases ER stress, and these responses are correlated with increased nitroso-redox imbalance.

### Nitric oxide supplementation could inhibit MYC and overcome ER stress responses in PCa cells

**Fig. 3:**
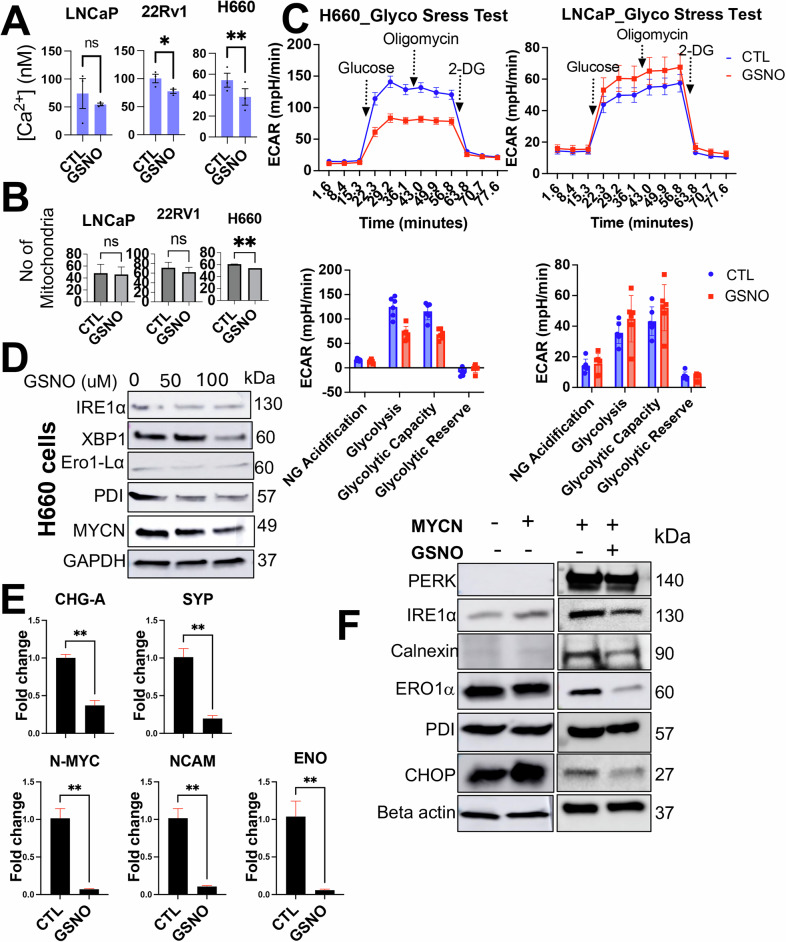
Nitric oxide supplementation inhibits MYC and inhibits ER stress responses in prostate cancer cells. **A** Bar graph showing overall calcium levels in LNCaP, 22Rv1, and H660 cells with or without GSNO treatment (50 μM) within 2 h of treatment. **B** Bar graph depicting the number of mitochondria in LNCaP, 22Rv1, and H660 cells with or without GSNO treatment (50 μM), assessed using mitochondrial dye. **C** Results of Seahorse XF Mito Stress Tests on LNCaP and H660 cells with or without GSNO treatment. The graph shows a double plot of Extracellular Acidification Rate (ECAR) from three independent biological replicates. **D** Western blot analysis showing the inhibitory effects of GSNO treatment on ER stress markers in H660 cells. GAPDH serves as a loading control. **E** Quantitative real-time PCR results demonstrating the inhibitory effects of GSNO treatment on neuroendocrine prostate cancer (NEPC) markers in H660 cells. Data represent mean ± standard deviation from three independent biological replicates (*p* < 0.001). **F** Western blot analysis showing the inhibitory effects of GSNO treatment on ER stress markers in MYCN overexpressing 22Rv1 cells. GAPDH serves as a loading control.

Under conditions of ER stress, the ER’s ability to regulate calcium homeostasis is compromised in neuroendocrine cells [33, 34]. As a result, abundant calcium ions could be released into the cytoplasm through channels such as inositol 1,4,5-triphosphate receptors (IP3Rs) and ryanodine receptors (RyRs). These elevated Ca^+2^ are taken up by the mitochondria, and excessive intake can further enhance the production of ROSs, which exacerbates oxidative stress and, therefore, adds to the mitochondrial dysfunction [35]. To confirm if MYC has a direct effect on Ca^+2^ release in high-grade PCa cells, we evaluated Ca^+2^ in 22Rv1 cells, which overexpresses MYCC and MYCN. Results showed that the 22Rv1 overexpressing MYCN showed a significant increase in the Ca^+2^ release comparable to the MYCC overexpressing or control (CaCl_2_ group) (Supplementary Fig. 4). Previous studies from our group have elucidated NO’s antioxidative properties in PCa cell lines [36]. Expanding on this foundation, we aimed to explore whether NO supplementation (using S-nitroso glutathione, an NO donor) could inhibit ER stress responses. In this context, first, we tested the inhibitory effects (if any) of NO treatment on reducing ER stress-induced Ca^+2^ release. For this, we used LNCaP, 22Rv1, and H660 cells treated with either vehicle or GSNO (50 μΜ), which were loaded with FURA-2. Subsequently, the fluorescence was acquired, and ratiometric data were analyzed. Results showed that the GSNO decreased the Ca^+2^ release in CRPC (22Rv1) and NEPC (H660) cells and not in primary PCa cells (LNCaP) comparable to the positive control (CaCl_2_ group) (Fig. 3A). Although we could not directly determine whether IP3R or RyR mediated this effect, both receptors share structural similarity and belong to the same intracellular calcium release channel family [37]. Both are known targets of S-nitrosylation by NO, which can modulate their calcium-release activity [38]. Thus, the observed inhibition of Ca²⁺ release may result from GSNO-mediated regulation of one or both channels. Further studies are needed to delineate their individual contributions.

To investigate NO’s downstream bioenergetic and metabolic impacts (if any) on NEPC cells, we treated LNCaP, 22Rv1, and H660 cells with GSNO (50 µM) over a 48-h period. Post-treatment, we evaluated the number of mitochondria in the cells (an indicator of cellular health and bioenergetics) using quantitative fluorescence microscopy, employing MitoTracker Green FM. Notably, we observed a significant decrease in the number of mitochondria in H660 cells (Fig. 3B). Similar patterns were observed when comparing MyCAP, MyC-CaP^shAR^ and MyC-CaP^APIPC^ cells (Supplementary Fig. 5). Additionally, we examined whether the observed decrease in mitochondrial count corresponded with changes in mitochondrial function. For this, we investigate the bioenergetic and metabolic impact of NO through Seahorse XF Mito Stress Tests on LNCaP and H660 cells. The cells were treated with 50 µM of GSNO for 48 h before subjecting them to the Seahorse test. This assay included sequential injections of 2 µM Oligomycin (to inhibit ATP synthase (complex V)) and 2 µM FCCP (Carbonyl cyanide 4-(trifluoromethoxy) phenylhydrazone) (to uncouple oxidative phosphorylation by dissipating the proton gradient, allowing for the measurement of maximal respiration) to measure mitochondrial function and the oxygen consumption rate (OCR). Results highlighted a significant reduction in glycolysis and glycolytic capacity in H660 cells alone. No significant impact was observed on mitochondrial respiration (basal respiration, maximal respiration, proton leak, ATP production, and spare respiratory capacity). A slight reduction was observed in non-mitochondrial oxygen consumption rate in H660 cells post-NO treatment compared to LNCaP cells (Fig. 3C).

To study the effects of NO supplementation against the ER stress at molecular levels, we monitored the expression of IRE1α, Ero1- Lα, PDI, and XBP-1, respectively, post-treatment of H660 cells with GSNO at 50 and 100μΜ concentrations. These UPR markers, displayed decreased expression levels upon GSNO treatment (Fig. 3D). Additionally, the expression of Chromogranin (CHG-A), Synaptophysin (SYP), MYCN, Neural cell adhesion molecule (NCAM) and Enolase (ENO) was diminished in the presence of NO in H660 cells (Fig. 3E). This observation suggests a downregulation of pathways associated with aggressive tumor behavior and hypoxic adaptation [39, 40], pointing to NO’s capacity to downregulate oncogenic signaling cascades. Furthermore, we subjected the MYCN overexpressing 22Rv1 cells to GSNO to determine if NO supplementation could overcome MYCN-induced ER stress response in high-grade PCa cells. Cells with enforced MYCN expression coupled with NO treatment exhibited reduced ER stress markers relative to their counterparts overexpressing MYCN without NO treatment (Fig. 3F). This delineates the inhibitory role of NO against the deleterious mitochondrial effects induced by MYCN overexpression. Together, these results underscore the potential therapeutic application of NO in attenuating MYCN-driven ER stress in high-grade PCa.

**Fig. 4:**
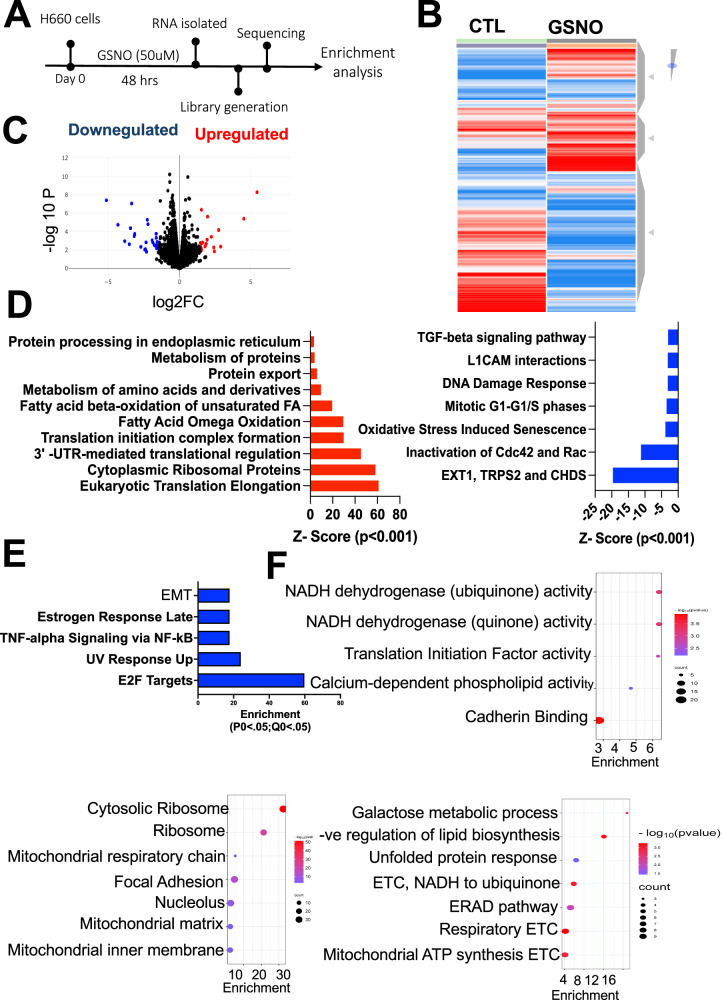
RNA sequencing reveals GSNO-induced transcriptional changes in H660 cells. **A** Schematic illustrating the RNA sequencing workflow for H660 cells treated with 50 μM GSNO for 48 h. **B** Heatmap depicting significantly differentially expressed genes (fold change > 2, *q*-value < 0.05) following 48-h GSNO treatment. **C** Visualization of differentially expressed genes (DEGs) with fold change > 1.5 and adjusted *P* < 0.05. Upregulated genes are shown in red, down-regulated genes in blue. **D** Bar graph highlighting key downregulated pathways, including ER stress signaling (featuring genes EXT1, TRPS2, CHDS, CDC42, and RAC), oxidative stress, cell cycle regulation, DNA damage response, L1CAM interactions, and TGF-beta signaling. **E** Bubble plot illustrating upregulated molecular processes. **F** Bubble plot showing enriched cellular processes. **G** Bubble plot depicting upregulated biological processes, including protein synthesis and metabolic pathways such as eukaryotic translation elongation, ribosomal protein synthesis, protein processing in the ER, fatty acid oxidation, and amino acid metabolism.

To elucidate the molecular landscape of NEPC cells and assess the effects of NO supplementation in greater detail, we treated H660 cells with 50 µM GSNO for 48 h, followed by RNA isolation and subsequent RNA sequencing (Fig. 4A–C). The enrichment analysis uncovered a significant downregulation of pathways associated with ER stress signaling, including genes such as EXT1, TRPS2, CHDS, CDC42, and RAC [41] (Fig. 4D, right). These pathways are integral to ER stress responses, and their downregulation indicates a reduction in the accumulation of misfolded proteins, which in turn enhances cellular homeostasis. This suggests that NO supplementation inhibits ER stress, likely by promoting the proper folding and processing of proteins within the ER. Additionally, key oxidative stress pathways linked to cellular senescence were markedly reduced by NO supplementation, consistent with decreased oxidative damage and improved stress resilience. These pathways are central to the progression of neuroendocrine differentiation and cancer aggressiveness. Further downregulated pathways included those involved in G1/G1-S cell cycle regulation, DNA damage response mechanisms [42], L1CAM interactions [36], and TGF-beta signaling [43] (Fig. 4D), pointing to a broad suppression of stress and proliferation-related pathways in NEPC cells. Simultaneously, pathways related to protein synthesis and metabolism were significantly upregulated following GSNO treatment (Fig. 4D). These included critical processes such as eukaryotic translation elongation, ribosomal protein synthesis, and 3’-UTR-mediated translational regulation [44], suggesting an enhanced capacity for protein synthesis. Furthermore, the initiation of translation complex formation [45], protein export [46], and protein processing in the ER [11] were also upregulated, indicating improved efficiency in managing ER load and preventing stress accumulation. This improvement in protein synthesis machinery suggests a more robust cellular response to meet the demands of protein folding and processing.

Moreover, Fig. 4E shows the pathways related to metabolic processes were enriched, with notable upregulation in fatty acid omega oxidation [47], mitochondrial fatty acid beta-oxidation of unsaturated fatty acids, and amino acid metabolism [47]. These enhancements reflect a shift toward greater metabolic activity, increased energy production, and lipid homeostasis. The improved balance in lipid metabolism would support membrane integrity and functionality in the ER, further contributing to the reduction in ER stress. Enhanced energy production, coupled with the increased availability of molecular building blocks for protein synthesis, suggests that GSNO supplementation supports a more efficient cellular environment capable of maintaining protein homeostasis. Together, these findings indicate that GSNO significantly modulates both ER stress and metabolic pathways in NEPC cells. The downregulation of stress-associated pathways and the simultaneous upregulation of protein synthesis and metabolism-related pathways suggest that NO supplementation via GSNO enhances cellular resilience, promotes protein homeostasis, and may attenuate the malignant progression of NEPC through these mechanisms.

### Exogenous induction of NO treatment demonstrates an inhibitory role against NEPC functions

**Fig. 5:**
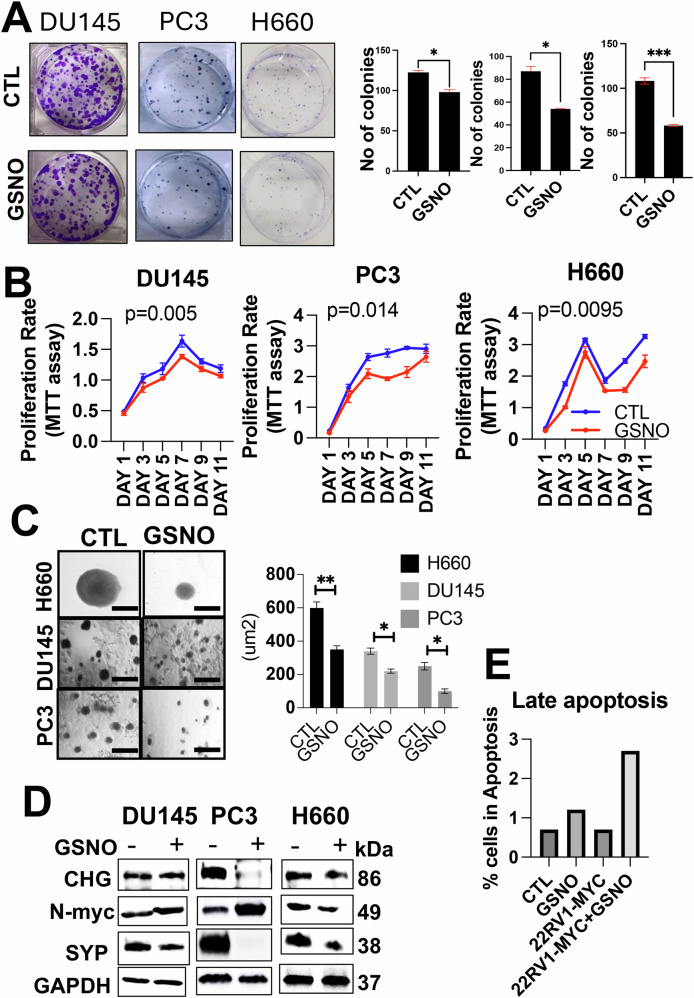
Exogenous induction of NO treatment demonstrates an inhibitory role. **A** bar graph showing the inhibitory effects of GSNO treatment on the colony-forming capabilities of DU145, PC3, and H660 cells. **B** Line graph illustrating the inhibitory effects of GSNO on cell proliferation (MTT assay) in DU145, PC3, and H660 cells over time. **C** effects of GSNO (50 μM) treatment on H660, DU145, and PC3 3D tumoroids upon 9 days of treatment. **D** Western blot results show neuroendocrine markers such as chromogranin, synaptophysin, and MYCN levels in PC3, DU145, and H660 protein lysates before and after GSNO treatment. **E** Bar graph demonstrating the pro-apoptotic effects of GSNO treatment on H660 cells and 22Rv1 cells overexpressing MYC. Data represent mean ± standard deviation from three independent biological replicates (*p* < 0.001, two-way ANOVA).

Next, we studied how NO treatment could disrupt the functional properties of high-grade PCa cells. For this, we treated PC3, DU145, and H660 cells with GSNO (50μM) for 48 h before seeding them for functional assays. Our results highlighted a suppression of colony formation in all the cell lines following NO treatment (Fig. 5A). In a similar context, MTT assays confirmed a significant inhibition of the proliferative capacity associated with NEPC (Fig. 5B). Furthermore, we evaluated the effects of GSNO treatment on tumor-like growth in 3D culture systems which more closely mimic the in vivo tumor microenvironment. We found that for PC3, DU145, and H660 cells cultured in 3D conditions, GSNO treatment at a concentration of 50 μM for 9 days significantly decreased tumoroid formation across all three cell lines, with the strongest effects in H660 cells (Fig. 5C). This reduction in tumoroid size and number suggests that NO signaling exerts inhibitory effects on PCa growth in a 3D environment, further highlighting its therapeutic potential.

As another measurement for the NO treatment’s effectiveness, we assessed the expression of NEPC markers, such as MYCN, CHG-A, SYP, and ER stress markers, such as PERK, CANX, BIP, and Ero1-L, in DU145, PC3 and H660 cells. Notably, H660 cells exhibited a marked downregulation of these markers after NO treatments, while PC3 cells followed a similar yet less pronounced trend and the DU145 cell line showed an even more moderate response. These data indicate that the sensitivity to NO treatment is likely specific to the NEPC phenotype (Fig. 5D and Supplementary Fig. 6A).

Moreover, we checked for the effects of NO in inducing apoptosis in H660 cells and in 22Rv1 cells, which stably overexpress MYC. The results highlighted an increase in overall late apoptosis upon NO supplementation in both cells (Fig. 5E). The concurrent decline in NEPC marker expression, inhibitory effects on colony formation, and increase in late apoptosis supports a disruptive role of exogenous NO on NEPC functions. Complementing this, we investigated the NO-mediated inhibitory impact on the transition dynamics of PCa, specifically from AR-dependent to AR-independent NEPC stages. For this, we subjected MyC-CaP, MyC-CaP^shAR,^ and MyC-CaP^APIPC^ cells to similar NO treatment. The MyC-CaP^APIPC^ and MyC-CaP^shAR^ cells displayed reduced colony-forming and cell proliferating capabilities (Supplementary Fig. 6B, C). Together, the results reveal that exogenous induction of NO treatment possesses a discernible inhibitory role against the functional properties of NEPC cells. The suppression of proliferative capacity and downregulation of NEPC-specific markers provide compelling evidence of NO’s therapeutic promise.

### Exogenous induction of NO demonstrates tumor inhibitory role against NEPC

**Fig. 6:**
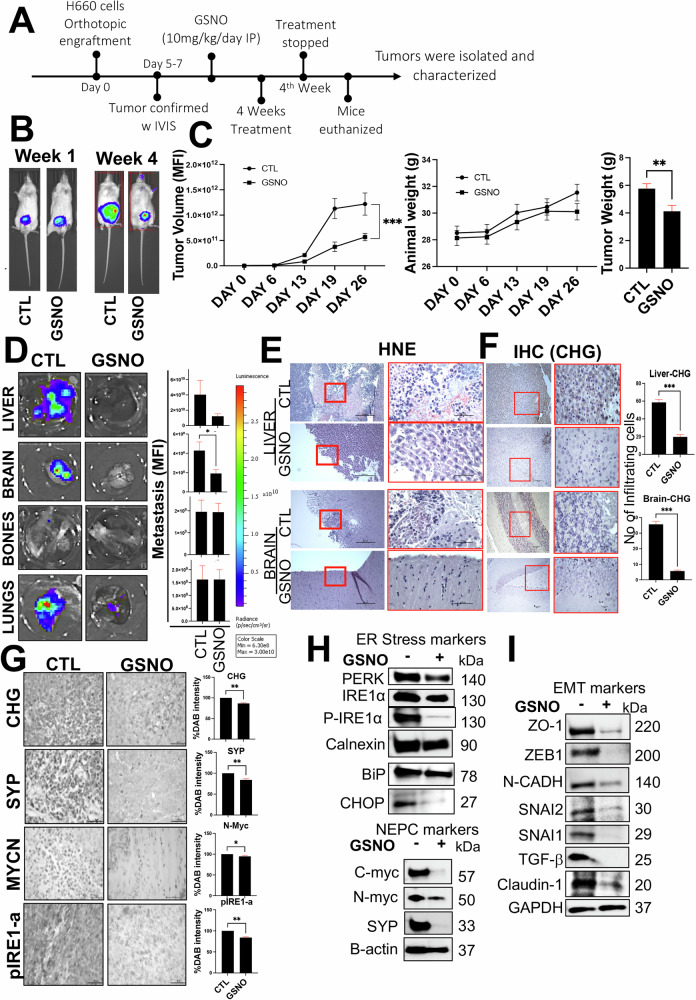
Exogenous Nitric Oxide Inhibits NEPC Tumor Growth and Metastasis. **A** Schematic illustrating the protocol used to evaluate the effects of NO stimulation on NEPC tumor burden. **B** Left: Representative bioluminescence imaging (BLI) of NOD-SCID mice (*n* = 8) orthotopically injected with H660-Luciferase tagged cells at week 0 and week 4. Right: Line graph showing mean tumor volumes over time for GSNO-treated and control groups. **C** Bar graphs depicting: Tumor volumes, Animal weights, Tumor weights in mice treated with or without GSNO (10 mg/kg/day, intraperitoneal). **D** Ex-vivo Imaging of Metastases**:** Left: Representative ex-vivo bioluminescence images of liver, brain, bone, and lung tissues highlighting metastatic areas. Right: Bar graph showing mean normalized photon flux/second (±SEM) from metastases in each tissue type. Student’s t-test, **p < 0.001. **E** Hematoxylin and eosin (H&E)-stained sections of liver and brain tissues. Arrows indicate metastatic lesions. **F** Images showing immunohistochemical staining for chromogranin (CHG) neuroendocrine markers in liver and brain tissue sections. **G** Images showing immunohistochemical staining for pIRE1-α, synaptophysin (SYP), and chromogranin A (CHG) in tumor sections from each group. Scale bar: 20 μm. **H** Western blot results showing NEPC and ER stress markers levels in tumor lysates from GSNO-treated and control mice. **I** Western blot results showing levels of EMT markers in tumor lysates from GSNO-treated and control mice.

To assess the impact of NO on NEPC tumor burden, we developed an orthotopic murine xenograft model using H660 cells [48]. The cells were transduced with CMV-Firefly luciferase lentivirus and selected using puromycin at a concentration of 2 μg/ml. Amplified cells were then orthotopically injected into the dorsal prostate of mice, as described by Shahryari et al. [49]. Each mouse received an injection of 3 million NEPC cells. Following injection, the mice were imaged using IVIS after luciferin injection (i.p.), with weekly imaging performed for four weeks to monitor tumor growth. GSNO was administered at 10 mg/kg/day intraperitoneally for the duration of the study. At the end of week 4, the mice were euthanized, and tumor volumes were estimated using mean fluorescent intensity (MFI) (Fig. 6A). The results indicated that GSNO treatment significantly reduced tumor growth (*p ≤ 0.05) compared to untreated control mice, as shown in Fig. 6B, C.

Additionally, GSNO treatment resulted in a significant reduction in metastatic colonization of the H660 cells to the brain and liver as assessed by bioluminescence imaging (BLI) and metastatic incidence (Fig. 6D). No metastasis was found in bones, and no significant reduction in lung metastasis (Fig. 6D, E). These findings are consistent with the metastatic progression in a clinical setting, as liver metastasis is associated with worse outcomes and neuroendocrine differentiation in patients [50]. Brain metastases, while rare in PCa overall, may be more common in NEPC due to its neuroendocrine and small cell-like features [51]. To test this, immunohistochemistry was performed using a chromogranin antibody on brain and liver sections derived from placebo and GSNO-treated mice. Results showed a significant reduction in chromogranin expression in GSNO-treated brains and liver (Fig. 6F), supporting the observations made in Fig. 6D. Next, western blot and immunohistochemistry were performed on the sections derived from tumor grafts of placebo controls and GSNO-treated mice. Results revealed a significant reduction in the expression of neuroendocrine markers MYCN, cMYC, Synaptophysin, Chromogranin, and ER stress markers such as PERK, BIP, pIRE1 alpha, PDI, and CHOP, respectively, in the GSNO-treated samples (Fig. 6G, H). These observations highlight the role of ER stress in NEPC transdifferentiation and, therefore, in delineating the metastatic landscape. To further evaluate the prognostic significance of ER Stress Markers in PCa metastasis, we performed Gene Expression Profiling Interactive Analysis (GEPIA). GEPIA showed that high expression of several ER stress markers Is associated with an increased risk of distant metastasis in men with PCa: MYCN: Hazard ratio (HR) for relapse = 2.1, p = 0.0018; DDIT3: HR for relapse = 2.3, p = 0.00019; PDI (Protein Disulfide Isomerase: HR for relapse = 2.0, p = 0.0012 and NOS3: HR for relapse = 1.7, p = 0.015 respectively. NOS3 involvement suggests a potential link between NO signaling dysregulation and metastasis (Supplementary Fig. 7). To confirm these observations, we evaluated the expression of markers of epithelial to mesenchymal transition in the protein lysates derived from murine tumor grafts from Fig. 6A. Results showed that NO supplementation, which was found to inhibit ER stress response markers, was able to significantly reduce the expression of markers such as TGFb, SNAI1, SNAI2, NCADH, ZEB1, Claudin, and ZO1, respectively (Fig. 6I). Together, these results indicate that higher expression of ER stress markers is associated with a greater risk of distant metastasis in high-grade PCa patients and NO supplementation can inhibit it.

**Fig. 7:**
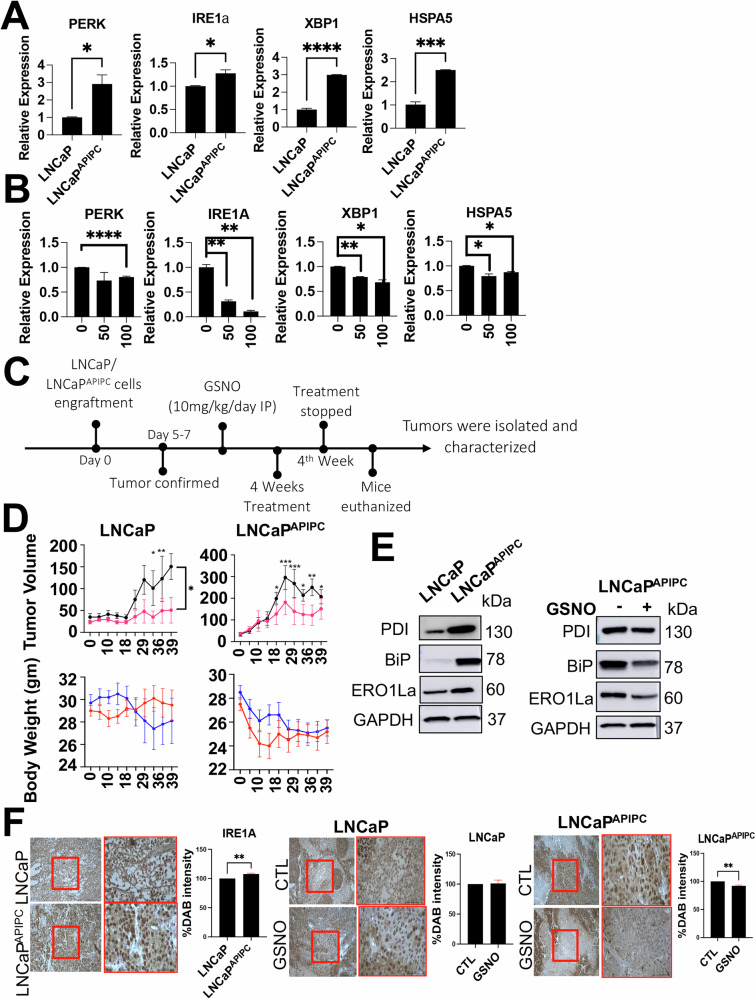
NO supplementation mitigates ER stress and inhibits tumor growth in AR-knockout prostate cancer models. **A** Comparison of ER stress marker expression (PERK, IRE1α, XBP1, and HSPA5) between LNCaP and LNCaPAPIPC cells, demonstrating significantly elevated expression in the AR-knockout (LNCaPAPIPC) model. **B** Gene expression analysis of ER stress markers following GSNO treatment (0, 50, and 100 µM) in LNCaPAPIPC cells, showing a dose-dependent reduction in expression. **C**, **D** Tumor burden analysis in NSG mice subcutaneously grafted with LNCaP (non-castrated) and LNCaPAPIPC (castrated) cells. Mice were treated with GSNO (10 mg/kg/day, IP) for four weeks, resulting in a significant reduction in tumor size in both groups. **E, F** Western blot and immunohistochemistry (IHC) analysis of tumor tissues from LNCaPAPIPC-grafted mice, revealing increased expression of ER stress markers (BiP, ERO1α, and PDI) in untreated tumors, which was significantly inhibited following GSNO treatment. Together, these findings reinforce the role of NO supplementation in modulating ER stress and reducing tumor burden in AR-deficient prostate cancer models, complementing observations from the NEPC model.

To further investigate the relationship between NO, ER stress, and tumor burden, we utilized an additional model representing complete AR loss, a hallmark of aggressive PCa progression. LNCaP^APIPC^ cells, derived from LNCaP with a complete AR knockout, were generously provided by Dr. Peter Nelson’s group. Given that AR loss is a key feature of therapy-resistant PCa, this model allowed us to examine whether NO’s ability to regulate ER stress and inhibit tumor growth is consistent in AR-negative prostate cancer cells. We first compared ER stress markers between LNCaP and LNCaP^APIPC^ cells. Results revealed a significant increase in PERK, IRE1α, XBP1, and HSPA5 expression in LNCaP^APIPC^ cells, confirming a heightened ER stress response in the absence of AR (Fig. 7A). Next, we exposed LNCaP^APIPC^ cells to 0, 50, and 100 µM GSNO and assessed the expression of the same ER stress markers. GSNO treatment led to a significant reduction in their expression (Fig. 7B), indicating NO’s ability to counteract ER stress in AR-deficient prostate cancer cells. To assess whether these molecular changes translate into tumor growth inhibition, we subcutaneously grafted LNCaP and LNCaP^APIPC^ cells into NOD scid gamma (NOD.Cg-Prkdc^scid^ Il2rg^tm1Wjl^/SzJ) mice (non-castrated for LNCaP and castrated for LNCaP^APIPC^). Once tumors became palpable, mice were treated with GSNO (10 mg/kg/day, IP) for four weeks. Tumor burden analysis revealed a significant reduction in both groups following GSNO treatment (Fig. 7C, D). Notably, LNCaP^APIPC^ tumors exhibited a pronounced increase in ER stress markers (BiP, ERO1α, PDI), reinforcing the association between AR loss and ER stress activation. However, GSNO exposure significantly inhibited these markers, as confirmed by Western blot and IHC (Fig. 7E, F). Together, these findings complement the results observed in the NEPC model, reinforcing the role of NO supplementation in mitigating tumor burden through ER stress modulation. While NEPC represents a distinct lineage plasticity-driven phenotype, the observation that NO similarly suppresses ER stress and tumor growth in AR-deficient LNCaP^APIPC^ tumors suggests that its therapeutic potential warrants further investigation in broader AR-independent PCa contexts.

### NO induces S-nitrosylation of MYCN to inhibit ER stress responses

**Fig. 8:**
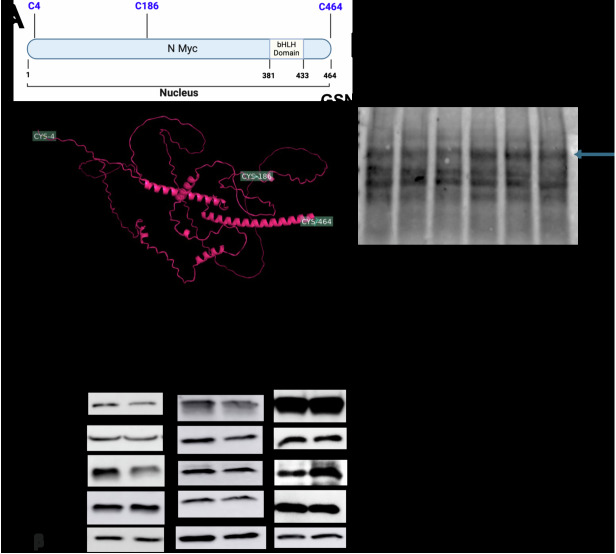
S-nitrosylation of MYCN by GSNO and its effects on protein binding and ER stress in NEPC cells. **A** GPS-SNO 1.0 software predicted 16 cysteine residues on the MYCN protein susceptible to S-nitrosylation, with Cys4, Cys186, and Cys464 exhibiting the highest thresholds for nitrosylation (cutoff 2.443). **B** Biotin-switch assay confirmed S-nitrosylation of MYCN upon GSNO treatment, specifically affecting the heavy chain of MYCN. **C** Western blot showing the protein expression of ER stress markers (PDI, BIP, and CHOP) in 22Rv1, DU145, and H660 cells, post-transfection with MYCN plasmid-containing mutations at Cys4, Cys186, and Cys464 sites, followed by GSNO treatment (50 μg) for 48 h.

One of the primary mechanisms by which NO exerts its cellular effects is through S-nitrosylation, a process wherein a nitroso moiety from NO-derived metabolites covalently attaches to a reactive cysteine residue, forming an S-nitrosothiol (SNO) [52, 53]. Given the significant inhibitory effects of NO supplementation on MYCN-induced ER stress, we hypothesized that S-nitrosylation of MYCN by GSNO (S-Nitrosoglutathione) is behind dampening the ER stress response. Using the GPS-SNO 1.0 software, we identified 16 cysteine residues on the MYCN protein, conforming to the acid-base nitrosylation conservative motif. Among these, Cys4, Cys186, and Cys464 showed the highest predicted thresholds for S-nitrosylation (cutoff 2.443) (Fig. 8A and Supplementary Fig. 8A). To confirm that GSNO induces S-nitrosylation of MYCN, we employed a biotin-switch assay on protein lysates derived from tumor samples of mice treated with or without GSNO, which demonstrated the formation of S-nitrosylated MYCN upon GSNO treatment, specifically in the heavy chain of MYCN (Fig. 8B). This highlights that NO supplementation can modify MYCN through S-nitrosylation, altering its function.

To investigate whether S-nitrosylation of MYCN at specific cysteine residues contributes to NO-mediated suppression of ER stress in NEPC, we generated site-directed mutations at Cys4, Cys186, and Cys464 (Supplementary Fig. 8B). Structural analysis of MYCN revealed the topological locations of these cysteines (Fig. 8A), which may influence MYCN’s functional domains. We introduced these mutated MYCN constructs into 22RV1, DU145, and H660 cell lines. Following transfection, cells were exposed to vehicle or GSNO (50 μM) treatment for 48 h, after which protein lysates were analyzed via western blot for the ER stress markers. The results (Fig. 8C) demonstrated that mutations at S-nitrosylation sites on MYCN prevented NO-induced ER stress inhibition in H660 cells, whereas no such effect was observed in 22RV1 or DU145 cells. These findings strongly suggest that NO specifically targets MYCN to suppress ER stress responses in NEPC, with S-nitrosylation at Cys4, Cys186, and Cys464 playing a crucial role in this selective regulatory mechanism.

**Fig. 9:**
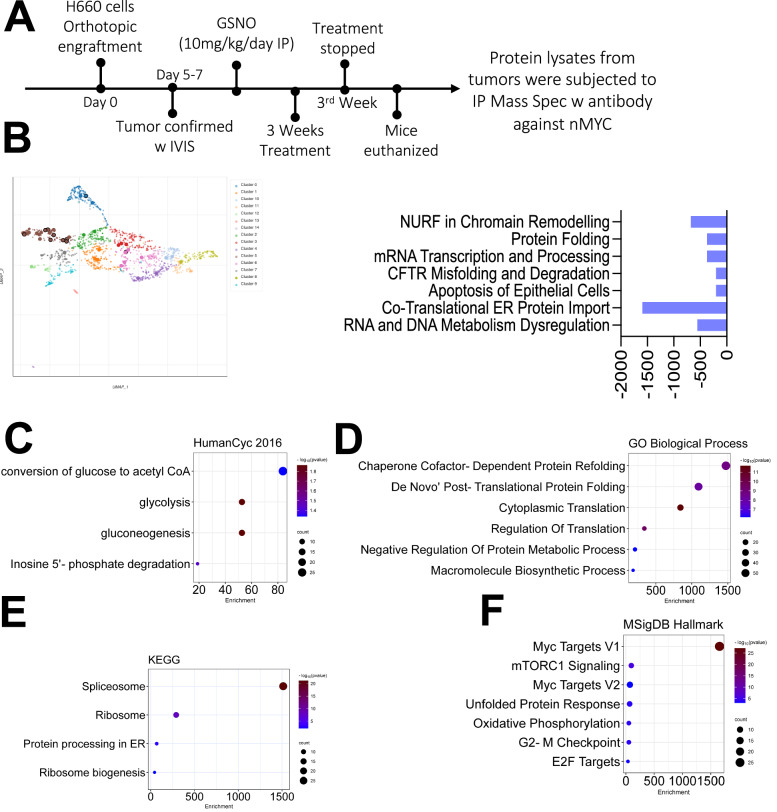
Proteomic analysis of GSNO treatment effects on protein interactions in NEPC tumors. **A** Schematic illustration of the immunoprecipitation followed by mass spectrometry (IP-MS) protocol used to analyze protein interactions in control and GSNO-treated mice. **B** Comparison of protein interactions between control and GSNO-treated mice. NO supplementation via GSNO inhibited the binding of proteins involved in key cellular processes such as RNA/DNA metabolism, ER protein import, Protein folding, and Chromatin remodeling. **C**–**F** Gene Ontology and pathway analyses were performed on down-regulated proteins in nuclear lysates from tumor and GSNO-treated tumor samples. The results are presented in bubble plots, where bubble size represents the number of genes and color intensity indicates statistical significance. **C** Human Cyt 2016: Bubble plot illustrating enriched cytological terms associated with down-regulated proteins. This analysis provides insights into the cellular compartments and structures affected by GSNO treatment. **D** GO Biological Process: Bubble plot showing enriched cellular processes impacted by GSNO treatment. This analysis highlights the biological functions most significantly altered in response to NO supplementation. **E** KEGG Pathway Analysis: Bubble plot depicting enriched cellular pathways affected by GSNO treatment. This analysis reveals the broader cellular systems and networks influenced by NO-mediated protein interaction changes. **F** MSigDB Hallmark: Bubble plot showing enrichment of hallmark gene sets, providing a high-level overview of the cellular states and processes altered by GSNO treatment.

We further hypothesized that NO-induced S-nitrosylation could interfere with the binding of various protein partners to MYCN, thus impairing its ability to promote tumor progression in NEPC cells. To explore this, we conducted proteomic analysis through IP-MS in GSNO-treated NEPC xenografts using antibody against MYCN (Fig. 9A). Analysis outcomes showed a substantial reduction in MYCN-binding proteins linked to RNA and DNA metabolism, ER protein import, CFTR misfolding and degradation, transcriptional regulation, and chromatin remodeling (Fig. 9B). Figure 9C–F details the spectrum of inhibited interactions, with notable declines in co-translational ER import processes and protein-folding functions. This pattern underscores a targeted disruption in MYCN’s capacity to maintain proteostasis within the ER, which is further corroborated by the decreased presence of protein partners involved in misfolding management and chromatin structure regulation. The data suggest that NO-induced S-nitrosylation of MYCN reconfigures the ER’s adaptive response, enhancing protein-folding efficiency while limiting the accumulation of misfolded proteins that typically contribute to stress. These modifications to ER function are clinically relevant, as they point to a reduction in the UPR threshold in NEPC cells, thereby lessening the cells’ ability to counterbalance stress-induced damage and maintain lineage plasticity. By compromising MYCN’s control over these cellular networks, NO treatment disrupts NEPC cells’ resilience and adaptability—a key factor in their survival and resistance to standard treatments. These findings highlight the therapeutic potential of targeting MYCN S-nitrosylation to destabilize the cellular equilibrium essential for NEPC progression, offering a strategic avenue for reducing therapeutic resistance in this aggressive cancer phenotype.

## Discussion

NEPC remains a formidable challenge in the clinical management of PCa due to its aggressive nature and resistance to conventional therapies [1, 2, 5, 6]. ER stress is a pivotal factor in NEPC progression, as it facilitates tumor cell survival under hostile metabolic conditions. MYCN is a well-known oncogenic driver of neuroendocrine differentiation [3, 4, 54–64]. However, its role in contributing to the increased biosynthetic demands of neuroendocrine cancer cells remains understudied. This information is essential because sustained ER stress leads to increased Ca^2+^ release, mitochondrial dysfunction, and the generation of ROS, which in turn exacerbates ER stress, creating a vicious cycle that promotes tumor growth and metastasis [9–12]. Understanding how MYCN, can play an essential role in delineating ER stress responses and other metabolic changes within an NEPC cell could assist in effectively targeting NEPC cancers. Our study sheds light on the molecular underpinnings of MYCN-driven ER stress and demonstrates how the dysregulation of a protein modification, S-nitrosylation allows the MYCN to increase the ER stress in an NEPC cell. We demonstrated that overcoming this limitation through exogenous supplementation of nitric oxide, a molecule whose production otherwise is reduced in high-grade PCa, can reinstate the S-nitrosylation of MYCN at specific sites (Cys4, Cys186, Cys46). The positive effects of NO treatment are observed not only in reducing the tumor burden of NEPC but also in reducing the overall metastasis to the Brain and Liver in murine models. This observation aligns with the findings of retrospective studies in human, which state that NEPC patients have significantly more frequent visceral metastases compared to patients with castration-resistant adenocarcinoma (62% vs 24%, *p* < 0.001) with Liver and Brain metastases, particularly common in NEPC [1, 65]. Furthermore, our in vitro studies elucidated how NO supplementation can reverse the effects of ER stress by the suppression of key unfolded protein response (UPR) markers, a survival mechanism to cope with increased protein folding demands and cellular stress; reducing the release of Ca^2+^ from ER to the cytoplasm, thereby inhibiting the overall glycolytic stress in the NEPC cells; ultimately inhibiting the colony forming and proliferative properties of NEPC cells.

The therapeutic implications of these findings are significant. Given the poor response of NEPC to standard chemotherapies and emerging chemo-immunotherapy combinations [1, 2, 5, 6], our data highlight NO supplementation as a promising therapeutic strategy. By targeting the nitroso-redox imbalance and restoring S-nitrosylation in NEPC cells, NO donors like GSNO could potentially overcome one of the key mechanisms of therapy resistance—namely, the unchecked activity of MYCN. This approach not only addresses the metabolic and biosynthetic imbalances driven by MYCN overexpression but also restores mitochondrial function and inhibits the ER stress that underpins NEPC aggressiveness. Moreover, the role of NO in inhibiting lineage plasticity offers a novel approach to halting the transition of PCa cells to a neuroendocrine phenotype. As lineage plasticity remains one of the most intractable problems in NEPC, disrupting this process through the modulation of MYCN function could pave the way for more effective treatment regimens.

While the results of our study provide robust evidence for the therapeutic potential of NO-based therapies in NEPC, there are several limitations to consider. First, the extent to which NO supplementation can be integrated into existing therapeutic regimens needs to be further explored. Combination therapies involving NO donors and current chemo-immunotherapies may offer synergistic benefits, but this hypothesis remains to be tested in clinical trials. Additionally, while our in vivo models showed promising reductions in tumor burden, the long-term effects of NO supplementation on NEPC progression, especially regarding potential resistance mechanisms, require further investigation. Furthermore, given that a broad range of proteins are subjected to S-nitrosylation, it is possible that other proteins besides MYCN may also contribute to the regulation of NEPC progression. Finally, the biological role of NO is not limited to S-nitrosylation. For example, the production of NO requires arginine, which is an important amino acid that plays multiple roles. For instance, arginine is also a required substrate for the reaction of arginylation, another type of posttranslational modification with known implications in regulating proteotoxic stress as well as the balance between glycolysis and mitochondrial activity [66–68]. Future research should focus on understanding the broader implications of S-nitrosylation in regulating other oncogenic drivers beyond MYCN. It is also essential to investigate the role of NO in modulating the tumor microenvironment (TME), particularly in terms of immune cell infiltration and the potential reprogramming of tumor-associated macrophages (TAMs), which could further enhance the anti-tumor efficacy of NO-based therapies.

In conclusion, our study uncovers a novel mechanism by which ER stress and MYCN-mediated lineage plasticity drive NEPC progression. The therapeutic potential of NO supplementation to restore S-nitrosylation, re-regulate ER stress responses, and inhibit the oncogenic activity of MYCN presents a promising avenue for addressing the treatment resistance and aggressiveness of NEPC. This approach may offer a new strategy for improving outcomes in patients with this challenging form of prostate cancer.

## Material & methods

### Transcriptomic data analysis and survival correlation

Human prostate cancer samples used in this study were derived from both institutional tissue repositories and publicly available datasets. For the institutional cohort, formalin-fixed, paraffin-embedded (FFPE) prostate tumor specimens were obtained from the University of Miami Tissue Bank. All samples were fully de-identified prior to use and acquired under standard institutional biospecimen policies. In accordance with federal guidelines (45 CFR 46.104(d) (4)), the use of these de-identified archival tissues did not require specific IRB approval or informed consent, as the study did not involve direct interaction with human subjects.

In addition to the in-house samples, transcriptomic and clinical data were retrieved from two large, publicly accessible datasets: The Cancer Genome Atlas Prostate Adenocarcinoma (TCGA-PRAD) cohort, comprising RNA-seq profiles from 500 patients, and the Decipher Genomic Resource Information Database (GRID), containing gene expression data from 4983 prostate cancer cases. All data from these sources were de-identified and utilized in accordance with the ethical and legal data-sharing guidelines defined by their respective repositories.

RNA-seq data from 500 patients in the TCGA PRAD cohort and 4983 patients from the Decipher GRID database were analyzed to investigate gene expression changes associated with PCa progression. Patients were stratified based on clinical parameters including Gleason scores (GS), age, PSA levels, and therapy history. The RNA-seq data were obtained in raw count format. Initial preprocessing involved normalization using the transcripts per million (TPM) method to account for differences in sequencing depth across samples. Variance-stabilizing transformation (VST) was applied to the normalized data to reduce heteroscedasticity and improve interpretability for downstream statistical analyses. Differential expression analysis was conducted using the DESeq2 package (version 1.26.0) in R. Raw read counts were modeled with a negative binomial distribution to estimate fold changes in gene expression between patient groups. Comparisons were made between patient subgroups based on Gleason scores and other clinical characteristics. The results of the differential expression analysis were filtered using a false discovery rate (FDR) of less than 0.05 to control for multiple testing, and only genes with a log2 fold change of ±1 or higher were considered for further analysis. Correlation analysis was performed to assess the relationships between gene expression profiles. Pearson correlation coefficients were calculated for selected genes across the Decipher GRID dataset. Prior to correlation analysis, the data were mean-centered and scaled. A two-tailed t-test was used to evaluate the statistical significance of the correlation coefficients, with *p*-values less than 0.05 considered significant. Correlation matrices were visualized using heatmaps generated with the ggplot2 package in R. Survival analysis was carried out to assess the clinical relevance of gene expression levels using the cBioPortal platform. Kaplan-Meier survival curves were generated to compare overall survival (OS) between patient groups stratified by gene expression levels (high versus low), which were determined based on the median expression of each gene. Hazard ratios (HR) with 95% confidence intervals were calculated using the log-rank test to determine the strength of the association between gene expression and survival outcomes.

### Cell culture

PC3 (CRL-2505), DU145 (CRL-1740) and H660 (CRL-5813) were purchased from ATCC. PC3 cells (AR^-^; NEPC-like) were maintained in F12K medium. DU145 cells (AR^-^; NEPC-like) were grown in DMEM medium. 22Rv1 cell line was cultured in RPMI-1640. NCI-H660 (CRL-5813)(NEPC cell line), were cultured in HITES medium. All cells but H660 (5%) were cultured in presence of 10% fetal bovine serum (Gibco), 100 U/ml penicillin, and 100 μg/ml streptomycin.

MyC-CaP cells (3 × 10^5^) were stably transfected with SMART shRNA doxycycline-inducible Lentiviral vector (Horizon Discovery Ltd.) to generate MyC-CaP^shAR^ cell lines. These cells were further modified via Lipofectamine 2000 (Invitrogen) transfection of a plasmid encoding a triple-probasin-driven herpes thymidine kinase (HSV-TK) and a puromycin resistance cassette, which was a gift from Peter Nelson’s lab. A clonal population of this cell line derived from puromycin (Invitrogen) selection and serial dilution in a 96-well plate, which we refer to as MyC-CaP ^shAR/pATK^, was subjected to total AR pathways suppression (TAPS): for two weeks the cells were grown in RPMI1640 + 10%CSS; at week 3, media was supplemented with 1 mg/mL doxycycline. Media was changed every 3–4 days and MyC-CaP^shAR/pATK^ was maintained under TAPS for five months. A surviving colony of proliferating cells emerged. Following a 3-month expansion, this population of cells was treated with 50 µM ganciclovir (GCV; InvivoGen) for two weeks to eliminate any cells still robustly expressing an AR transcriptional program. We referred to the surviving population as MyC-CaP^APIPC^.

### MTT assay

PC3, DU145 and H660 cells (5000 cells per well) were seeded in 96-wells plate in quadruplets and were treated with varying doses of S-nitrosoglutathione (GSNO). MTT (3-(4,5-dimethylthiazole)-2,5-diphenyltetrazolium bromide, Sigma Aldrich CT01) assay reagents were added, and the absorbance was measured at 562 nm after 0, 3, 5, 7, and 9 days.

For RNA/protein collection, when cells were around 80% confluent, growth media was changed by those using charcoal stripped FBS. Then, treated with GSNO 50μM for different time points. After treatment, a colony formation assay using 2000 cells/well was done and stained with Cristal Violet, and cells were collected for RNA/protein isolation.

### Preparation of RNA and quantitative real-time PCR

Total RNA was extracted from cells using the Trireagent (Sigma-Aldrich T9424) method and then reverse transcribed to complementary DNA using High-Capacity cDNA Reverse Transcription Kits (Applied Biosystems, USA) according to the manufacturer’s protocol. The quantitative RT-PCR for indicated genes (MYCN, CHGA, SYP, ENO2, NCAM1, Ki67) was performed in SYBR Universal PCR Master Mix (BioRad 1725274). Quantitation of mRNAs was performed using BIORAD™ Gene Expression Assays according to the manufacturer’s protocol. Samples were analyzed using the BIORAD sequence detection system. All PCRs were performed in triplicate, and the specificity of the reaction was determined by melting curve analysis at the dissociation stage. The relative quantitative method was used for the quantitative analysis. The calibrator was the average ΔCt from the untreated cells. The endogenous control was glyceraldehyde 3-phosphate dehydrogenase (GAPDH).

### Western blotting

For Western blotting, cells were lysed using RIPA buffer (Thermo Fisher, #89900) supplemented with Protease Arrest™ (G-Biosciences, 786-450) and Phosphatase Arrest™ (G-Biosciences, 9806) inhibitor cocktails. Protein concentrations were measured using the BCA Protein Assay Kit (Thermo Fisher Scientific, 23225). Equal amounts of protein (20–40 µg) were separated by SDS-PAGE on 10–12% polyacrylamide gels and transferred to PVDF membranes (Millipore, IPVH00010) using wet transfer at 100 V for 1 h at 4 °C. Membranes were blocked with 5% non-fat dry milk in TBS-T for 1 h at room temperature and incubated overnight at 4 °C with primary antibodies diluted 1:500–1:1000. The following antibodies were used: N-Myc (Cell Signaling, 51705S; Abcam, ab24193), CHGA (Abcam, ab45179), SYP (Abcam, ab32127), EZH2 (Cell Signaling, [catalog number to be confirmed]), ER stress markers (BIP, Calnexin, Ero1-Lα, IRE1α, CHOP, PERK, PDI; Cell Signaling, 9956), androgen receptor (Abcam, ab133273), and GAPDH (Abcam, ab181603; Cell Signaling, 2118S; Santa Cruz Biotechnology, sc-47724). XBP-1S and P-IRE1α antibodies were in-house reagents. After washing, membranes were incubated with HRP-conjugated secondary antibodies (anti-mouse IgG, Cell Signaling, 7076S; anti-rabbit IgG, Cell Signaling, 7074S) for 1 h at room temperature. Protein bands were visualized using the SuperSignal™ West Pico PLUS Chemiluminescent Substrate (Thermo Scientific, 34580) and imaged using a Bio-Rad ChemiDoc system. Densitometric analysis was performed using ImageJ software, with GAPDH used for normalization.

### Immunohistochemistry

For immunohistochemistry, tissue sections were stained with hematoxylin and eosin and analyzed by a genitourinary pathologist. For immune-histochemical staining, tumor xenograft tissues were fixed in 10% buffered formalin and embedded in paraffin. Five-micrometer-thick sections were deparaffinized and rehydrated in sequential xylene and graded ethanol. Antigen retrieval was performed in 10 mM citrate buffer (pH 6.0) in a microwave oven. Peroxidase and non-specific protein blocking were done as per the instructions using the Abcam ABC detection kit (ab64264) and incubated with the following primary antibody dilutions: CHG (Cell Signaling, 70076), SYP (Abcam, ab178945) and MYCN (Cell Signaling, 4370) with 1:150 dilution. They were subsequently incubated with biotinylated goat anti-polyvalent secondary antibody, followed by development using DAB substrate as per the instructions on the kit. All sections were lightly counterstained with hematoxylin and mounted with Cytoseal XYL. Images were taken using a brightfield microscope (Nikon E200) at ×10 and ×40 magnification, and quantification was done using ImageJ 1 Front Endocrinol (Lausanne).

### Agonistic and antagonistic assays

22Rv1 cells were stably transduced with MYC viruses (PLV-10005, Cellomics) at a concentration of 1 × 10^8^ TU/ml. After addition of viruses, we waited for 48 h for cells to be infected. Puromycin selection antibiotic was used at a concentration of 5 μg/ml to select effectively transduced cells. Post selection, the remaining cells were allowed to grow further to at least 3 passages to use it for any further experiments.

### S-Nitrosylation assay

We evaluated if the post-translational modification S-nitrosylation, induced by GSNO, is acting as a degradation signal for MYCN. To do that we performed the biotin-switch assay following the manufacturer’s guidelines (S-Nitrosylated Protein Detection Kit (Biotin Switch), Item No. 10006518, Cayman Chemical, Ann Arbor, MI, USA). A small piece of tumor tissue or cell pellet was taken and washed twice with Wash Buffer. The pellets were resuspended in “Buffer A containing Blocking Reagent” and incubated for 30 min at 4 °C with shaking. The incubated samples were centrifuged, and the supernatant was transferred to 15 ml centrifuge tubes. Two milliliters of ice-cold acetone were added to each sample, and the mixture was incubated at −20 °C for at least 1 h. The protein from each sample was pelleted by centrifugation. “Buffer B containing Reducing and Labeling Reagents” was added to resuspend the proteins, with incubation for 1 h at room temperature. The biotinylated protein was precipitated by acetone and rehydrated with the appropriate amount of Wash Buffer. A total of 50 μg of protein was used for labeling and running the standard western blot for detecting the nitrosylated protein.

Besides, we deleted the S-nitrosylated sites in this protein by site-directed mutagenesis. MYCN ORF clone was procured from GenScript Biotech (NJ, USA). Site-directed mutagenic changes were performed to incorporate 3 cysteine deletions at C4, C186, and C464 (as determined by GPS-SNO 1.0 software with a high threshold) using Quick Change Lightning Multi Site-Directed Mutagenesis Kit (Agilent Technologies, USA), as previously described. Briefly, 40 ng plasmid was subjected to PCR amplification as per standard kit guidelines using mutagenic primers designed specifically for cysteine deletions at these specific sites. Following PCR, 10 μl of the product was subject to DpnI digestion for 5 min at 37 °C and transformed into chemically competent DH5α cells (NEB, USA) by heat shock at 42 °C for 30 s. The resulting transformants were grown in SOC media for 1 h at 37 °C and selected overnight on LB agar plates containing 100 μg/ml of Ampicillin. The following day, single colonies were selected and further grown. Plasmid isolation and purification were done using a plasmid miniprep kit (Qiagen, Germany) as per standard instructions. Sanger sequencing for confirmation of deletion was done by Genewiz, USA. For verification of these deletions, the confirmed wild-type, as well as mutant clones, were transfected in NCI-H660 cells using Lipofectamine 3000 reagent. After transfection, cells were treated with/without 50 µM GSNO, and cells were collected after 48 h to do western blots using specific antibodies for ER stress.

### Animals

The animal protocol was approved by the Institutional Animal Care and Use Committee of the University of Miami Miller School of Medicine, Miami, FL. SCID (3 weeks old) mice (B6.Cg-*Prkdc*^*scid*^/SzJ) were purchased from Jackson Laboratories. Castration experiments were performed in all the mice (which were used for experiments involving androgen-independent prostate cancer cells) when they reached the age of six weeks. Mice were housed in pathogen-free conditions, under a 12-h light/dark cycle, with ad libitum access to food and water. For castration, mice were anesthetized using Isoflurane (Abbott Laboratories). The perineal region was cleaned with ethanol and a betadine scrub (VWR, AJ159778), and sterile dissecting shears were used to make a 4–5 mm incision. Using two sterile forceps, the testes were located, and a ligature was made around the testicular vessels and the tunica albuginea that encases the testes. The testes were amputated with dissecting shears, and the scrotum was sutured closed with 6-0 Ethicon black monofilament nylon (Ethicon Inc., 1665). A local triple antibiotic was applied over the region of the wound to facilitate healing. SCID mice were grouped into control and experimental groups. The mice were grafted with 1 million NCI-H660 cells (orthotopically) or LNCaP and LNCaP^APIPC^ cells (subcutaneously). The mice were distributed randomly into the control and experimental group. The experimental group received 10 mg/kg/day of GSNO treatment intraperitoneally (IP) 3 times/week for 2 weeks, while the control group received PBS IP [18, 69]. Each group had 10 mice each. After treatment, animals survived for an additional two weeks before being humanely sacrificed. For this, euthanasia was performed via carbon dioxide asphyxiation with a displacement rate of 20–30% chamber volume per minute, followed by cervical dislocation as a confirmatory measure. Animals were monitored continuously during the procedure to ensure loss of consciousness prior to the secondary method. Isoflurane was also used for anesthesia prior to terminal procedures such as cardiac puncture. Following confirmation of deep anesthesia, blood was collected via terminal cardiac puncture using a 25 G needle into EDTA-coated syringes and processed immediately for downstream applications. Post euthanasia, tumor grafts, lungs, and spleen were harvested for further analysis. Tumor volume (V) was measured regularly (blinded) until the mice were sacrificed by measuring the length (L) and width (W) of the tumor with calipers by using the formula: *V* = 1/2(length × width^2^). Tumor growth was also monitored by IVIS.

### IP-mass spectrometry analysis

Tissue lysates were co-immunoprecipitated (co-IP) using MYCN antibody using Thermo Scientific™ Pierce™ Classic Magnetic IP/Co-IP Kit as per the standard guidelines mentioned in the kit. The MYCN ab was first added to the sample to form an immune complex that is then bound to the magnetic beads. The complex was washed to remove non-bound material and a low-pH elution buffer dissociated the bound immune complex from the Protein A/G. The beads were removed from the solution manually using a magnetic stand.

The eluted peptide mixtures were analyzed using a nanoflow liquid chromatograph (U3000, Dionex, Sunnyvale, CA) coupled to an electrospray ion trap mass spectrometer (LTQ-Orbitrap, Thermo, San Jose, CA) in a data-dependent manner for tandem mass spectrometry peptide sequencing experiments. Both MASCOT and SEQUEST search results were summarized in Scaffold 2.0. The integrated peak areas for phosphotyrosine peptide quantification were calculated from extracted ion chromatograms (EIC) using QuanBrowser from Xcalibur 2.0.

### GSNO reductase (GSNOR) activity assay

Cell lines were washed, trypsinized and homogenized by sonication (30’ in ice) in a solution containing 20 mM Tris-HCl (pH 8.0), 0.5 mM EDTA, 0.1% NP-40 and 1 mM phenylmethylsulphonyl fluoride (PMSF). To detect GSNO reductase enzymatic activity, 80 μg total protein was incubated with reaction buffer (20 mM Tris-HCl, pH 8.0, 0.5 mM EDTA) with 0 (negative control) or 200 μM NADH in the presence of GSNO 50 μM. Then, GSNO reductase activity was measured by reading absorbance at 340 nm every 5’ during 30’ (Nature, 410, 490–494, Liu 2001).

### Calcium release estimation

To assess the impact of nitric oxide (NO) supplementation on ER stress-induced calcium release in prostate cancer cell lines, we used a fluorescence-based approach. LNCaP (primary prostate adenocarcinoma), 22Rv1 (castration-resistant prostate cancer), and H660 (neuroendocrine prostate cancer) cells were cultured in RPMI 1640 media supplemented with 10% FBS and 1% penicillin–streptomycin at 37 °C with 5% CO2. Cells grown on a monolayer and treated with either 50 µM S-nitroso glutathione (GSNO) or vehicle, were loaded with 2.5 µM Fura-2 AM (Molecular Probes, Eugene, OR), a dual excitation calcium-sensitive dye, for 20 min at room temperature in PBS, followed by a 30-minute de-esterification period in the dark. Then, fluorescence (Ex: 340/380 nm, Em: 515 nm) was recorded in an IonOptix spectrofluorometer (IonOptix LLC, Westwood, MA). The calibration was performed on the cells acquiring fluorescence data in a Ca^2+^-free and then a Ca^2+^-saturating (5 mmol/L) solutions, both containing 10 μmol/L ionomycin (Sigma, St. Louis, MO) until reaching a minimal (R_*min*_) or a maximal (R_*max*_) ratio value, respectively. Intracellular Ca^2+^ ([Ca^2+^]i) was calculated using the following equation:

$${\boldsymbol{[}}{{{\boldsymbol{Ca}}}^{{\bf{2}}+}{\boldsymbol{]}}}_{{\boldsymbol{i}}}={K}_{d}\times \frac{{S}_{f2}}{{S}_{b2}}\times \frac{(R-{R}_{\min })}{({R}_{\max }-R)}$$

K_*d*_ (dissociation constant) in adult myocytes was taken as 224 nmol/L. The scaling factors S_*f2*_ and S_*b2*_ were extracted from calibration as described by Dulce et al. [70].

### Measurement of Oxygen consumption rate and Extracellular acidification rate

Oxygen consumption rate (OCR) and Extracellular acidification rate (ECAR) were measured using the Seahorse XF Pro Analyzer (Agilent Technologies). Mitochondrial stress test was performed to measure OCR per manufacturer’s instructions. Briefly, 4 × 10^4^ H660 or 3 × 10^4^ LNCaP cells were plated in complete growth media into each well of a 96-well Seahorse microplate and incubated overnight in 5% CO_2_ at 37 °C. Cells were then treated with GSNO for 48 h. Following GSNO treatment, cells were washed twice, incubated (in non-CO_2_ incubator at 37 °C for 1 h), and analyzed in XF assay media (non-buffered RPMI containing 10 mM glucose, 2 mM L-glutamine, and 1 mM sodium pyruvate, pH 7.4) at 37 °C, under basal conditions and in response to 2 μM oligomycin (Sigma), 2 μM fluoro-carbonyl cyanide phenylhydrazone (FCCP) (Sigma) and 0.5 μM rotenone (Sigma)/0.5 μM antimycin A (Sigma). Data were analyzed by the Seahorse XF Cell Mito Stress Test Report Generator. OCR (pmol O_2_/min) values were normalized to the protein content.

ECAR values were measured by performing glycolysis stress test according to manufacturer’s instructions. Briefly, 4 × 10^4^ H660 or 3 × 10^4^ LNCaP cells were plated in complete growth media into each well of a 96-well Seahorse microplate and incubated overnight in 5% CO_2_ at 37 °C. Cells were then treated with GSNO for 48 h. Following GSNO treatment, cells were washed twice, incubated (in non-CO_2_ incubator at 37 °C for 1 h), and analyzed in XF assay media (non-buffered RPMI containing 2 mM L-glutamine, pH 7.4) at 37 °C, under basal conditions and in response to 10 mM glucose (Sigma), 2 μM oligomycin (Sigma), and 50 mM 2-deoxy-D-glucose (Sigma). Data were analyzed by the Seahorse XF Cell Glycolysis Stress Test Report Generator. ECAR (mpH/min) values were normalized to the protein content.

### Three-Dimensional (3D) Tumoroid Culture Assay

Three-dimensional (3D) cultures were performed to evaluate tumor-like growth under physiologically relevant conditions. PC3, DU145, and NCI-H660 cells were seeded in ultra-low attachment 6-well plates (Corning, Cat# 7007) at a density of 2000 cells per well in 100 µL of growth medium containing 2% Matrigel (Corning, Cat# 356231). Cells were incubated under standard conditions (37 °C, 5% CO₂), allowing spontaneous formation of spheroids. GSNO (50 μM) or PBS (vehicle control) was added to the medium at the time of seeding, and treatments were refreshed every 3 days. After 21 days of culture, tumoroid growth was assessed by brightfield microscopy. Images were acquired using an EVOS FL Auto Imaging System (Thermo Fisher), and tumoroid number and size were quantified using ImageJ software. Tumoroid diameter was calculated as the mean of the longest and shortest axis. Experiments were performed in triplicate and repeated independently three times.

### Nitrite Determination (Griess Kit, Molecular Probes G7921)

Nitrite levels were quantified using the Griess Reagent Kit (Molecular Probes, G7921) according to the manufacturer’s instructions. Briefly, supernatant was collected from T75 flasks containing cells grown to confluency. The cells were cultured in the appropriate media, and once confluency was reached, the supernatant was harvested for analysis.

To measure nitrite, 150 µL of the collected supernatant was mixed with 150 µL of the Griess reagent in a 96-well plate, in triplicate, for each sample. A standard curve was prepared using known concentrations of sodium nitrite ranging from 0 to 100 µM in the same 96-well format. The plate was incubated at room temperature for 30 min, protected from light, allowing the color to develop.

Absorbance was measured at 548 nm using a microplate reader (insert model here), and the nitrite concentration in the samples was calculated by comparing the absorbance values to the standard curve. All measurements were performed in triplicate, and results were expressed as mean ± standard deviation.

### Statistical Analysis

All statistical analyses were conducted using R (version 4.0.3) and associated packages, unless otherwise specified. Data are presented as mean ± standard deviation (SD) or mean ± standard error of the mean (SEM), depending on the experiment. For comparisons between two groups, statistical significance was assessed using unpaired two-tailed Student’s t-tests. For comparisons involving more than two groups, one-way or two-way analysis of variance (ANOVA) was employed, followed by post hoc Tukey’s or Bonferroni correction for multiple comparisons where applicable. *P*-values less than 0.05 were considered statistically significant. All experiments were performed with at least three independent biological replicates unless otherwise indicated. Statistical analyses were performed using R or GraphPad Prism (version 9.0), and figures were generated using appropriate visualization tools such as ggplot2 and GraphPad Prism. A *p*-value of <0.05 was considered statistically significant across all analyses.

## Supplementary information

Supplementary Data

Original Blots

## Data Availability

All data, codes, and materials used in the study are available to any researcher for reproducing or extending the analysis. Materials transfer agreements (MTAs) will be required to request access. RNA sequencing data have been deposited in a public database. Further requests for information should be directed to and will be fulfilled by the lead contact, Himanshu Arora (Hxa287@med.miami.edu) and Rehana Qureshi (Rxq58@med.miami.edu).
