# Supplementary material for: Targeting endoplasmic reticulum stress and nitroso-redox imbalance in neuroendocrine prostate cancer: the therapeutic role of nitric oxide
Source: Cell Death Discov. 2025 Nov 6;11:502. doi: 10.1038/s41420-025-02774-5 (PMC12592524; doi:10.1038/s41420-025-02774-5)
Supplement: Supplementary file 1 — Supplementary Data [file 41420_2025_2774_MOESM1_ESM.docx]

**Supp. Figure 1.** The expression of ER stress markers in PCa patients' data was obtained from the Cancer genome atlas data (TCGA).

**Supp. Figure 2.** (a) Experimental steps are taken to generate MyCaP, MyCaPshAR, and MyCaPAPIPC cells, respectively. (b) shows expression of AR and MYCN in MyCaP, MyCaP^shAR^ and MyCaP^APIPC^ cells.

**Supp. Figure 3.** Griess test results showing nitrate concentrations in LNCaP and H660 cell lines to estimate nitrosative stress.

**Supp. Figure 4.** Bar graph showing overall calcium levels in 22Rv1 cells overexpressing MYCC or MYCN compared to control 22Rv1 cells. Data represent mean ± standard deviation from three independent biological replicates (p < 0.001, two-way ANOVA)

**Supp. Figure 5.** Results showing the number of mitochondria in the MyCaP, MyCaP^shAR^ and MyCaP^APIPC^ cells (an indicator of cellular health and bioenergetics) using quantitative fluorescence microscopy, employing MitoTracker Green FM.


**Supp. Figure 6.** (a) showing the inhibitory effects (if any) of GSNO treatment (50uM) on the ER stress markers in DU145, PC3 and H660 cells. (b) Shows the inhibitory effects of GSNO on the colony-forming and (c) cell proliferating capabilities of MyCaP, MyCaP^shAR^ and MyCaP^APIPC^ cells.

**Supp. Figure 7.** (a) shows the Gene Expression Profiling Interactive Analysis (GEPIA) of CHOP, NOS3, PDI, and MYCN, respectively, to evaluate the role of ER stress markers in distant metastasis in high-grade PCa patients.

**Supp. Figure 8.** (a) Showing the outcomes of GPS-SNO 1.0 software, which identified a total of 16 cysteine residues on the MYCN protein, conforming to the acid-base nitrosylation conservative motif. Among these, Cys4, Cys186, and Cys464 showed the highest predicted thresholds for S-nitrosylation (highlighted as yellow). (b) Sanger sequencing data to confirm the site-directed mutations at Cys4, Cys186, and Cys464.
