# Supplementary material for: Targeting endoplasmic reticulum stress and nitroso-redox imbalance in neuroendocrine prostate cancer: the therapeutic role of nitric oxide
Source: Cell Death Discov. 2025 Nov 6;11:502. doi: 10.1038/s41420-025-02774-5 (PMC12592524; doi:10.1038/s41420-025-02774-5)

FIGURE 2A

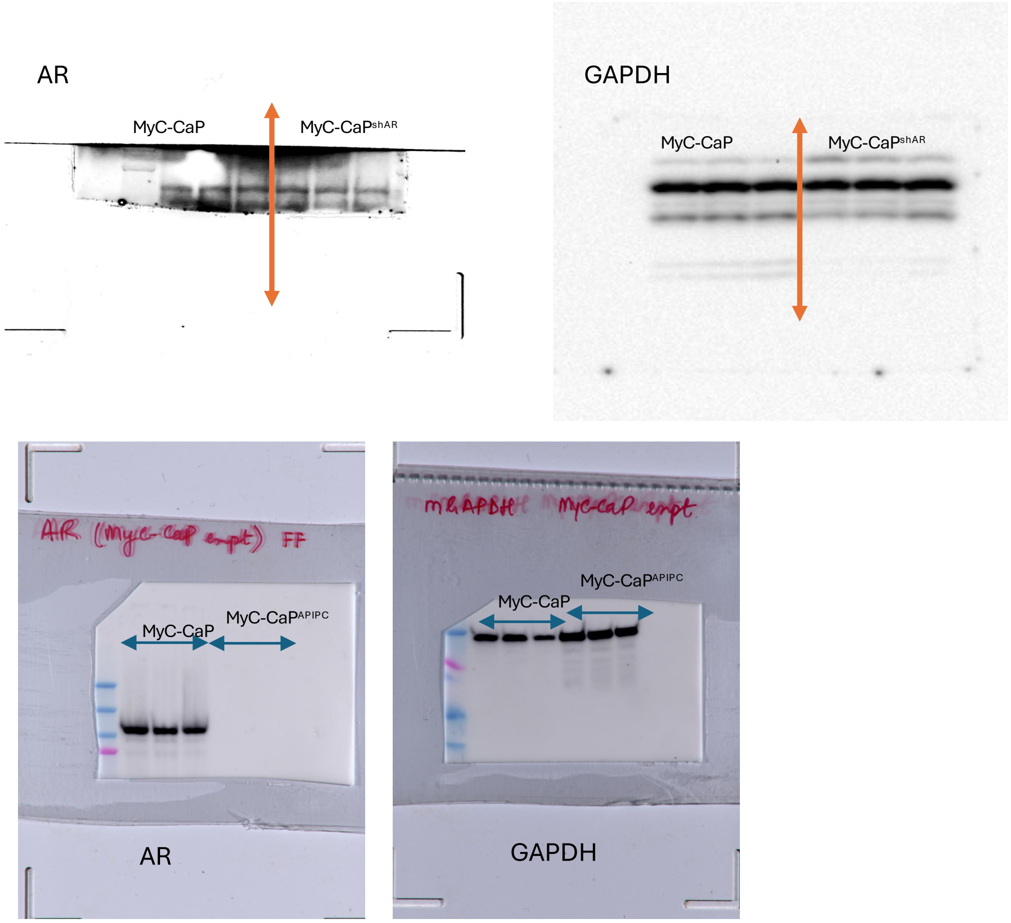

FIGURE 2C

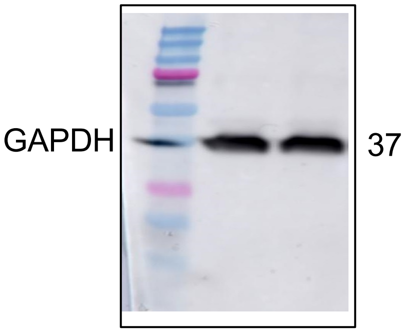

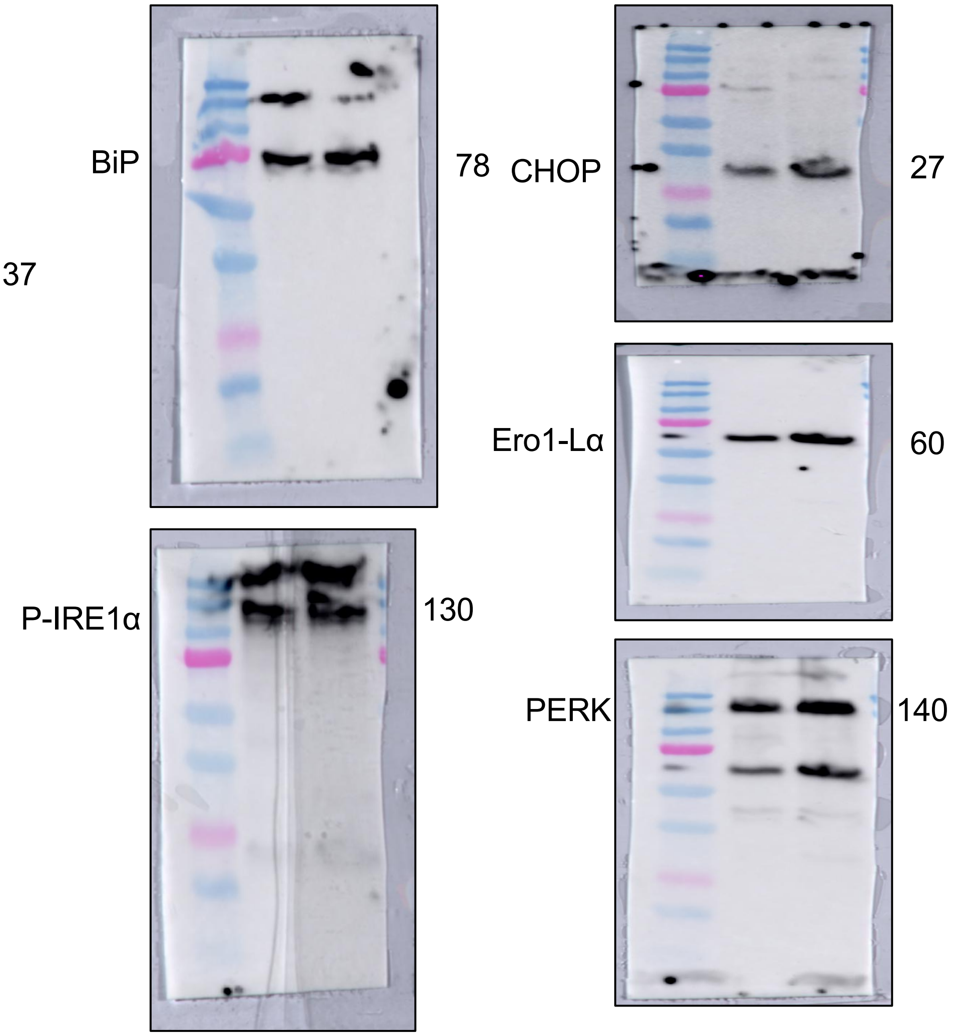

FIGURE 2D

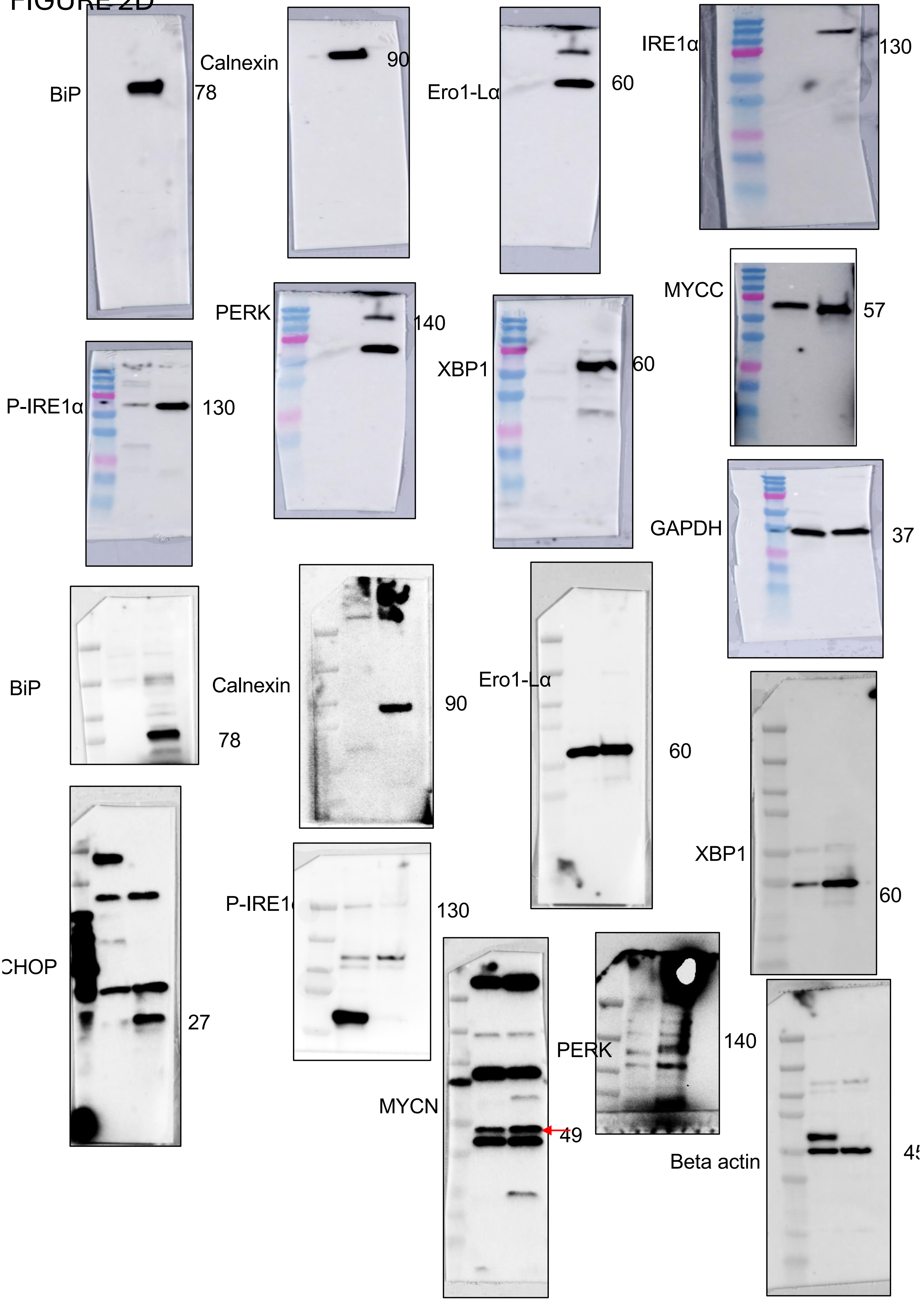
BiP
78
Calnexin
90
Ero1-Lα
60
IRE1α
130
PERK
140
P-IRE1α
130
XBP1
60
MYCC
57
GAPDH
37
BiP
78
Calnexin
90
Ero1-Lα
60
XBP1
60
27
P-IRE1
130
MYCN
49
PERK
140
Beta actin

FIGURE 3D

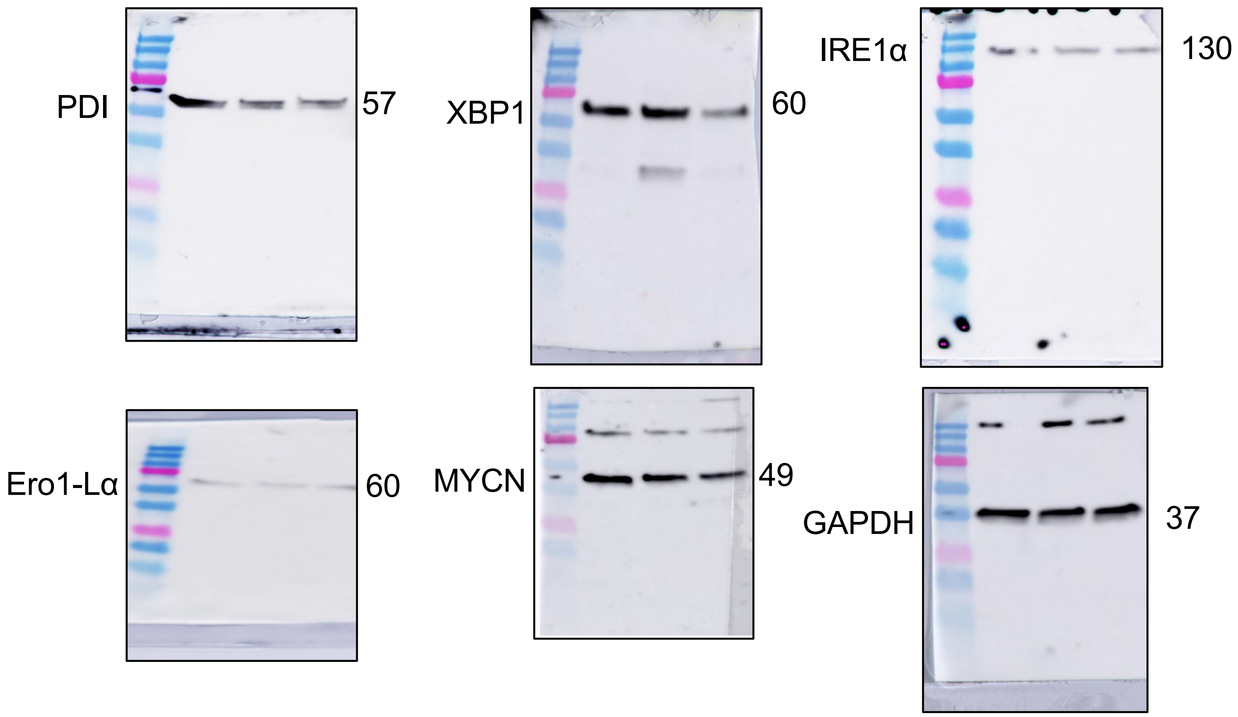

FIGURE 3F

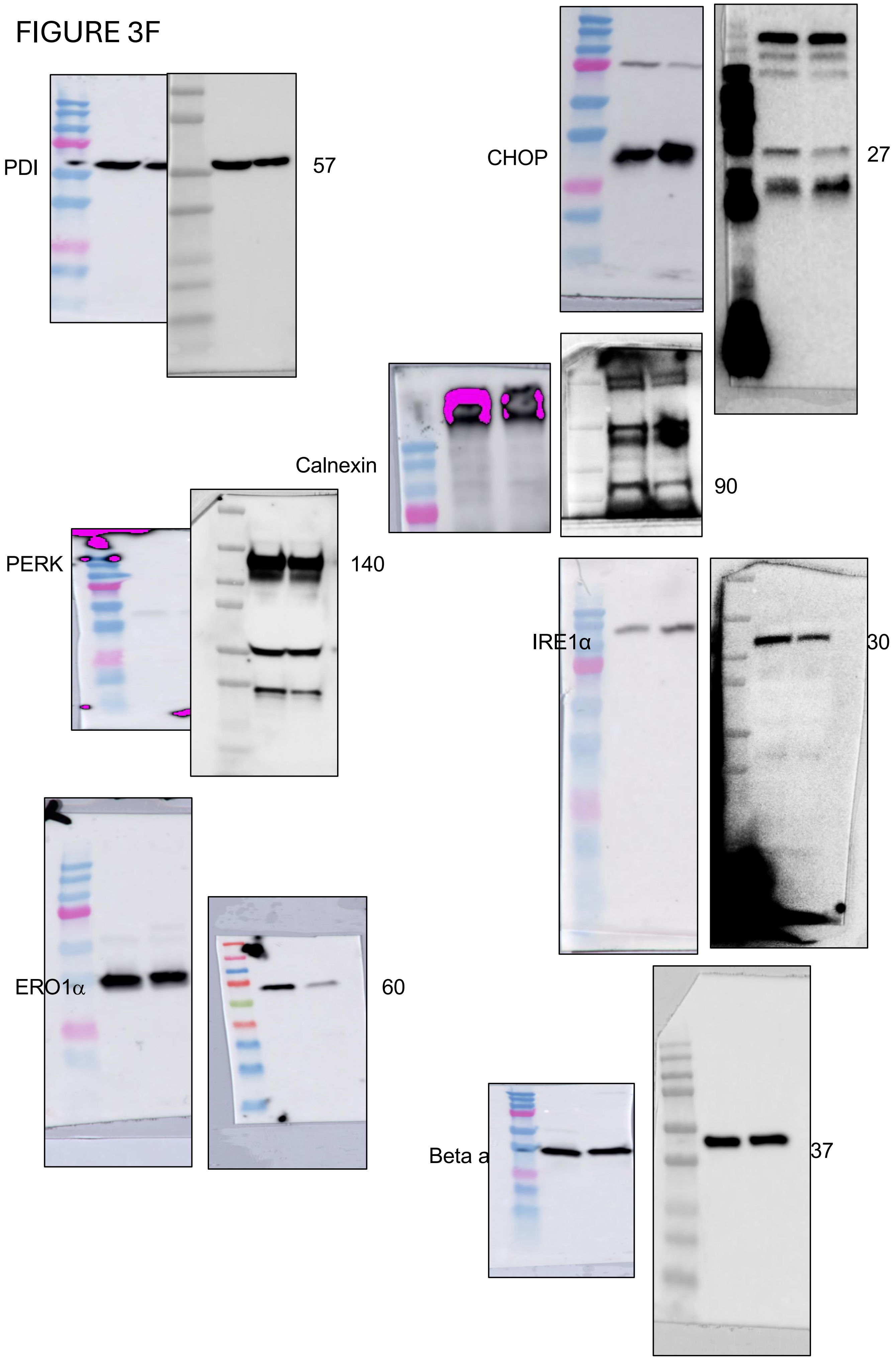

FIGURE 5D

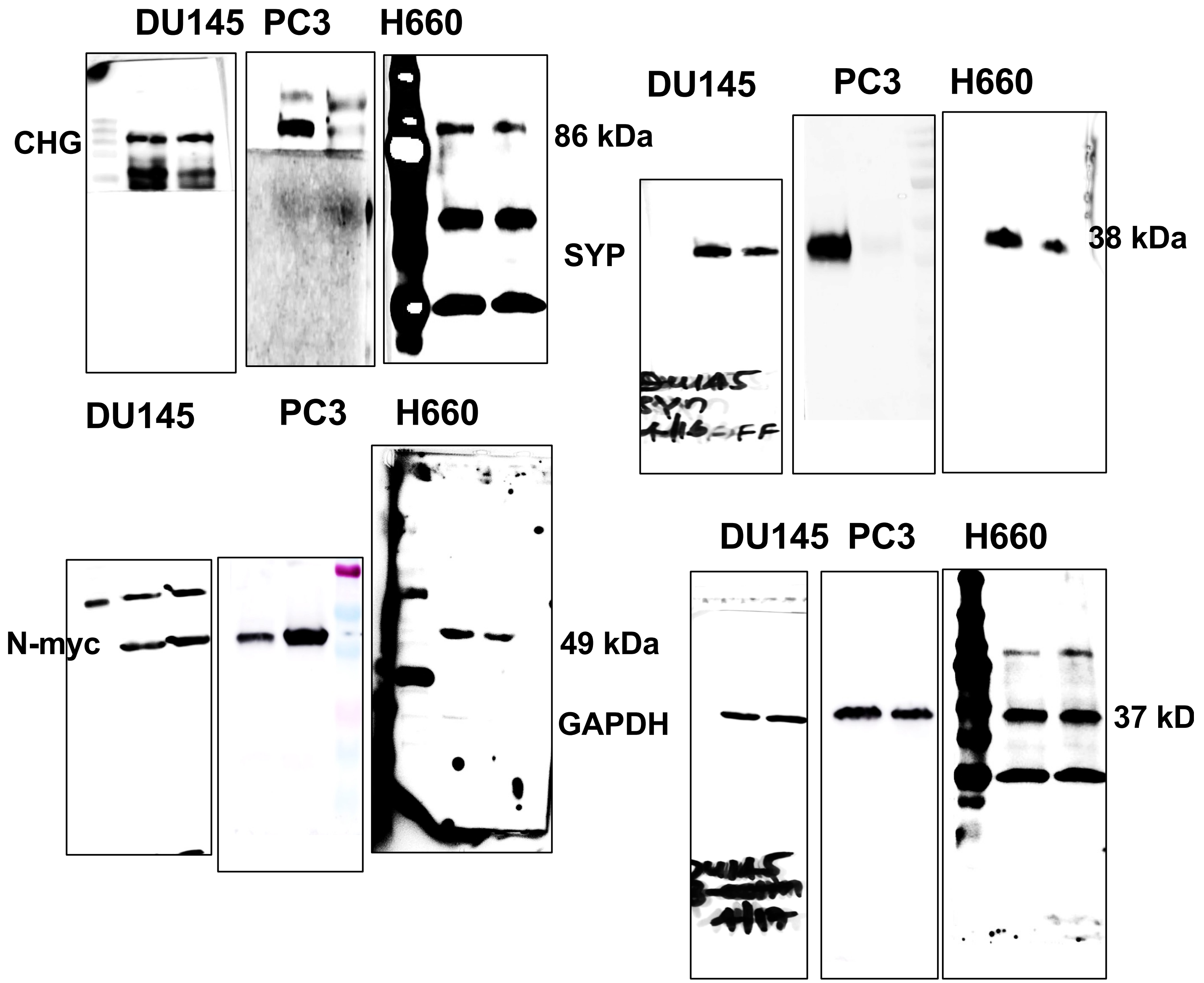
DU145
PC3
H660
CHG
86 kDa
DU145
PC3
H660
SYP
38 kDa
DU145
PC3
H660
N-myc
49 kDa
GAPDH
DU145
PC3
H660
37 kD

FIGURE 6H-Left panel

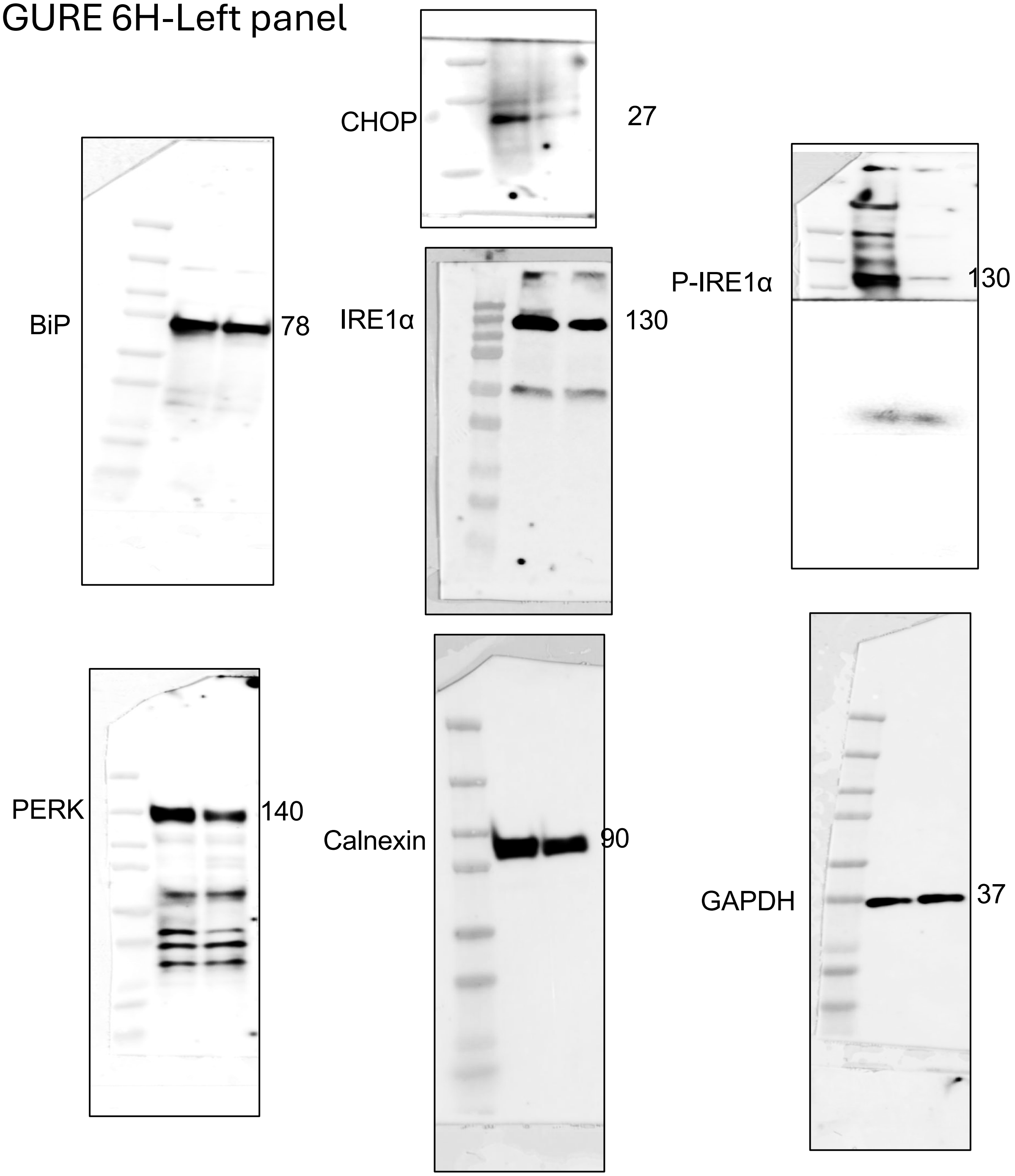

FIGURE 6H- middle panel

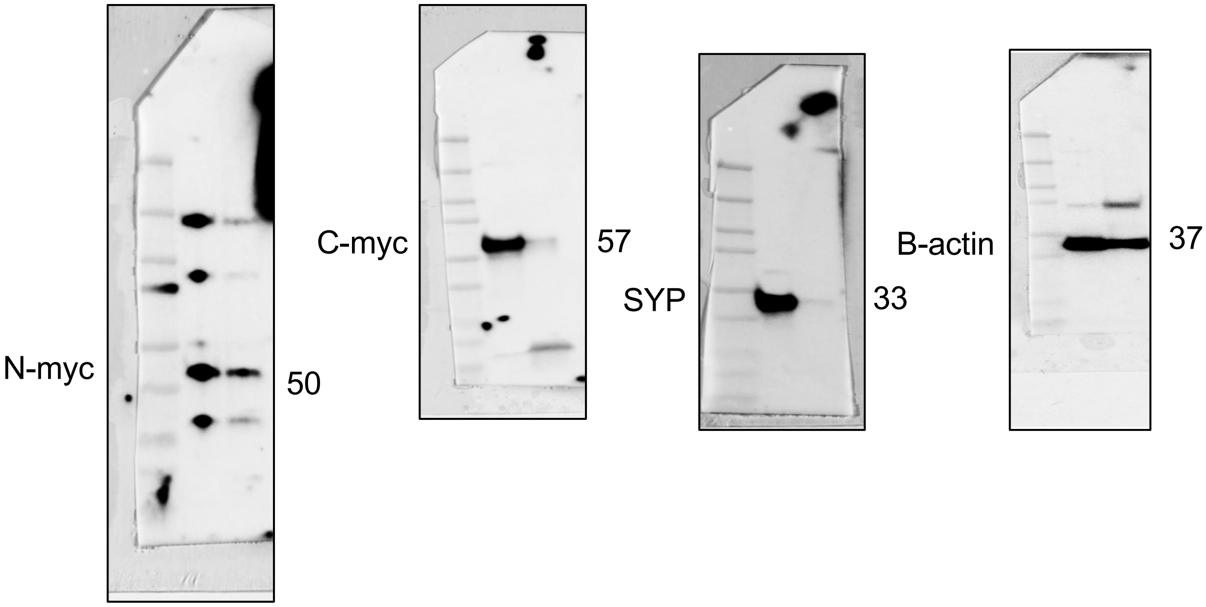

FIGURE 6H- right panel

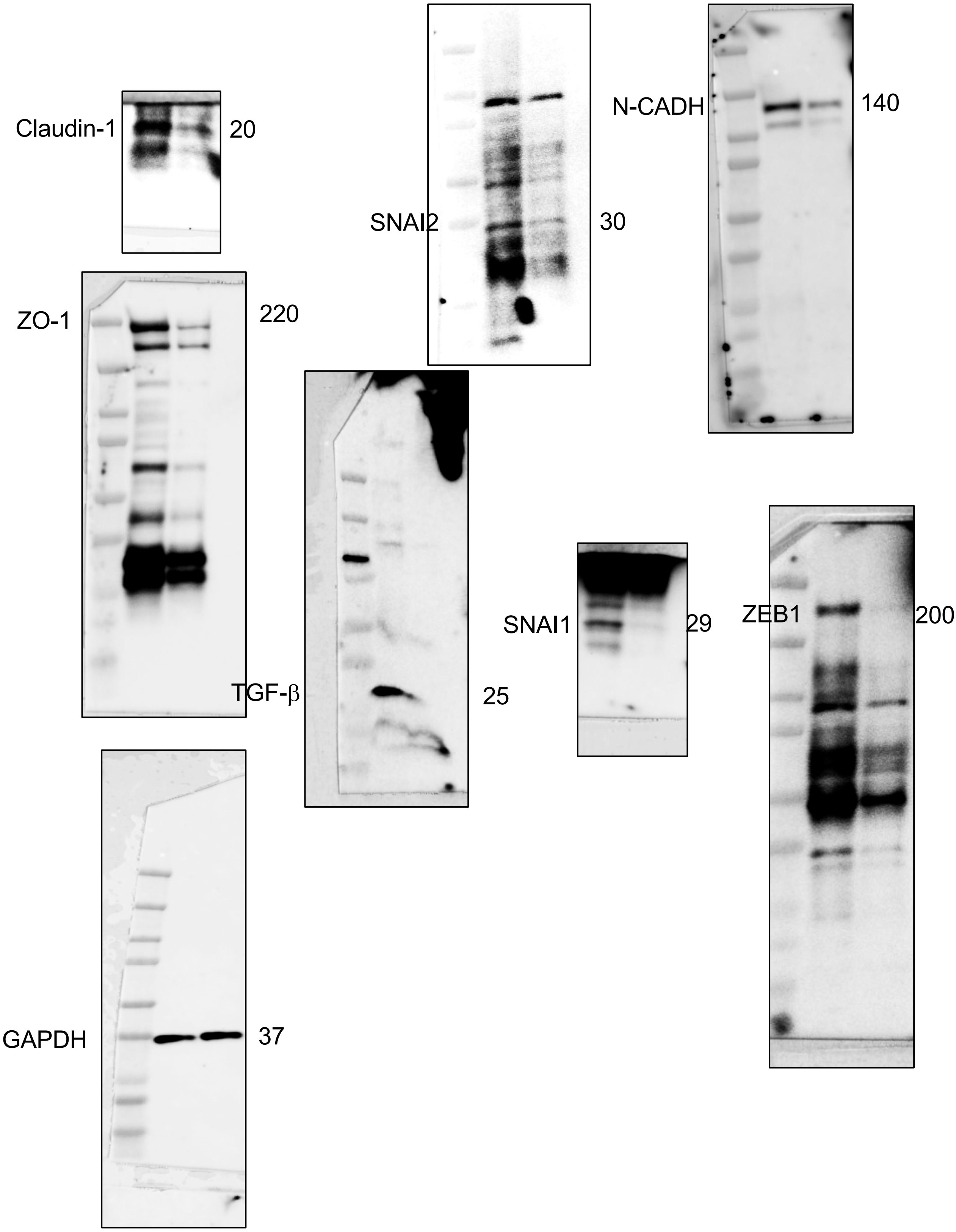

FIGURE 7E

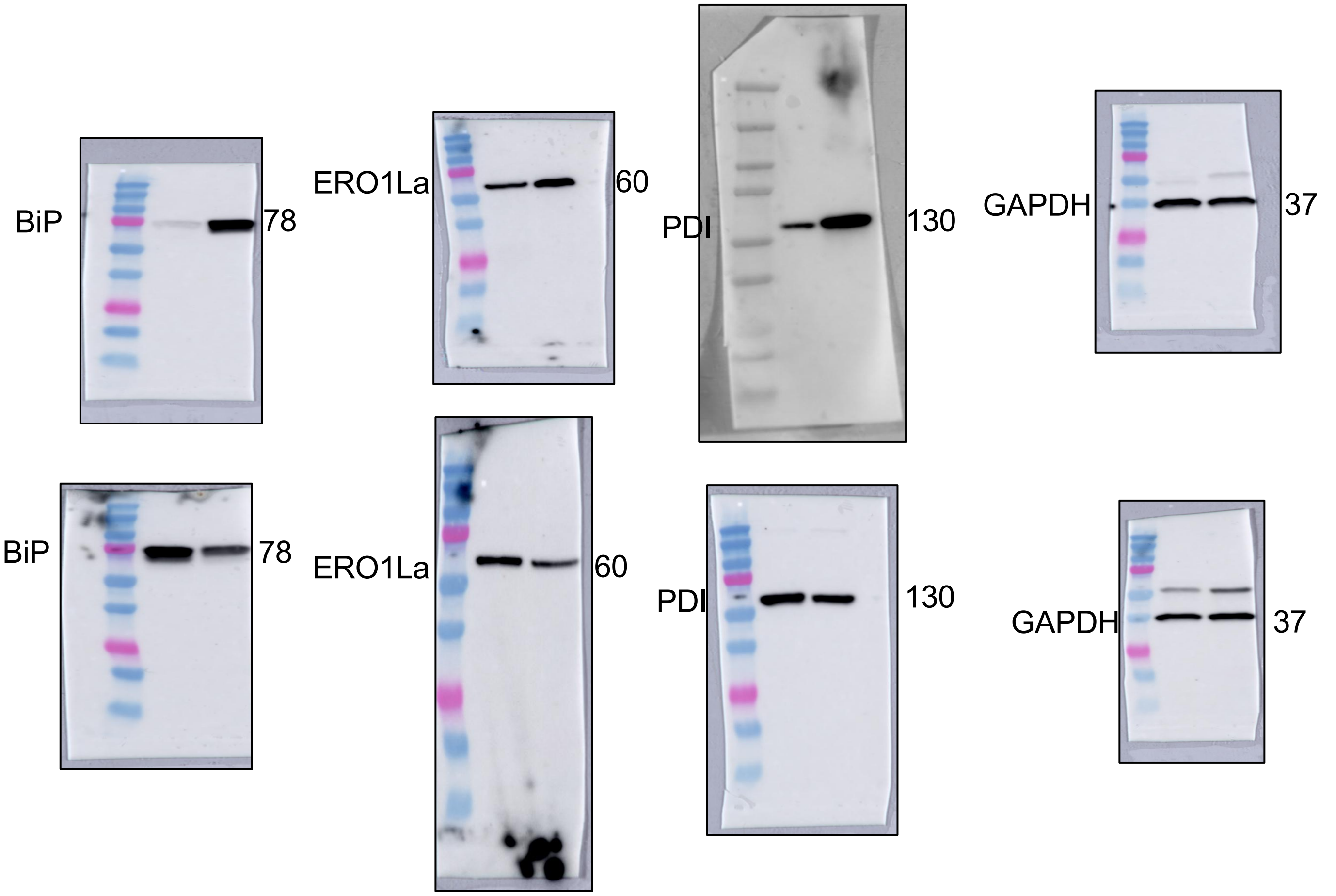
BiP
78
ERO1La
60
PDI
130
GAPDH
37
BiP
78
ERO1La
60
PDI
130
GAPDH
37

FIGURE 8C

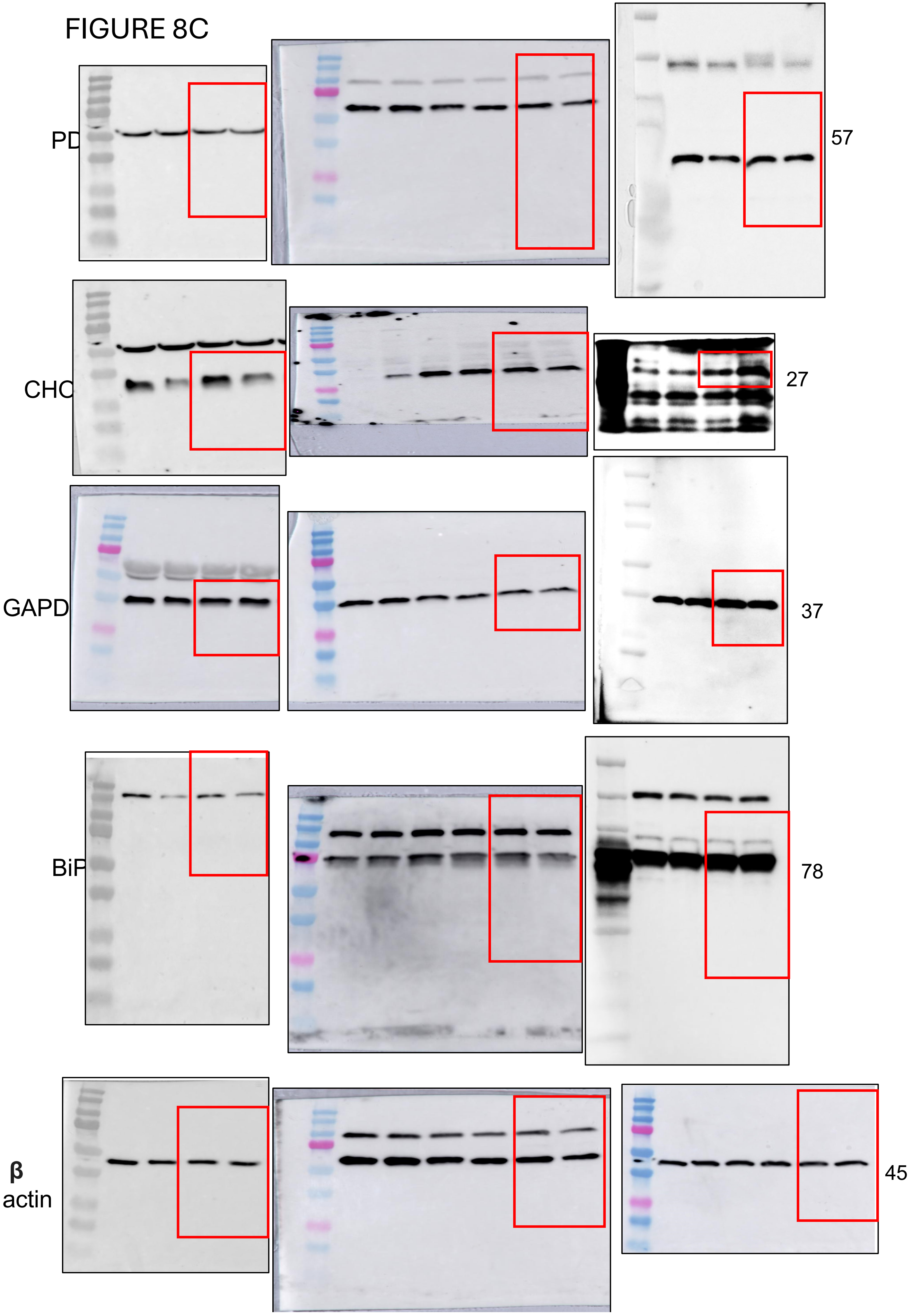

Supplement: Supplementary file 2 — Original Blots [file 41420_2025_2774_MOESM2_ESM.pdf]
